# Export of Diverse and Bioactive Small Proteins through a Type I Secretion System

**DOI:** 10.1128/aem.00335-23

**Published:** 2023-04-20

**Authors:** Sun-Young Kim, Jennifer K. Parker, Monica Gonzalez-Magaldi, Mady S. Telford, Daniel J. Leahy, Bryan W. Davies

**Affiliations:** a Department of Molecular Biosciences, The University of Texas at Austin, Austin, Texas, USA; b John Ring LaMontagne Center for Infectious Diseases, The University of Texas at Austin, Austin, Texas, USA; University of Michigan—Ann Arbor

**Keywords:** recombinant-protein production, protein export, type I secretion system, Gram-negative bacteria, type I secretion

## Abstract

Small proteins perform a diverse array of functions, from microbial competition, to endocrine signaling, to building biomaterials. Microbial systems that can produce recombinant small proteins enable discovery of new effectors, exploration of sequence activity relationships, and have the potential for *in vivo* delivery. However, we lack simple systems for controlling small-protein secretion from Gram-negative bacteria. Microcins are small-protein antibiotics secreted by Gram-negative bacteria that inhibit the growth of neighboring microbes. They are exported from the cytosol to the environment in a one-step process through a specific class of type I secretion systems (T1SSs). However, relatively little is known about substrate requirements for small proteins exported through microcin T1SSs. Here, we investigate the prototypic microcin V T1SS from Escherichia coli and show that it can export a remarkably wide range of natural and synthetic small proteins. We demonstrate that secretion is largely independent of the cargo protein’s chemical properties and appears to be constrained only by protein length. We show that a varied range of bioactive sequences, including an antibacterial protein, a microbial signaling factor, a protease inhibitor, and a human hormone, can all be secreted and elicit their intended biological effect. Secretion through this system is not limited to E. coli, and we demonstrate its function in additional Gram-negative species that can inhabit the gastrointestinal tract. Our findings uncover the highly promiscuous nature of small-protein export through the microcin V T1SS, which has implications for native-cargo capacity and the use of this system in Gram-negative bacteria for small-protein research and delivery.

**IMPORTANCE** Type I secretion systems for microcin export in Gram-negative bacteria transport small antibacterial proteins from the cytoplasm to the extracellular environment in a single step. In nature, each secretion system is generally paired with a specific small protein. We know little about the export capacity of these transporters and how cargo sequence influences secretion. Here, we investigate the microcin V type I system. Remarkably, our studies show that this system can export small proteins of diverse sequence composition and is only limited by protein length. Furthermore, we demonstrate that a wide range of bioactive small proteins can be secreted and that this system can be used in Gram-negative species that colonize the gastrointestinal tract. These findings expand our understanding of secretion through type I systems and their potential uses in a variety of small-protein applications.

## INTRODUCTION

Engineering microorganisms to secrete recombinant proteins into the extracellular space enables the discovery of new protein functions, efficient production, and investigation of sequence-activity relationships and routes for *in vivo* delivery (1–4). As a host for extracellular secretion, the Gram-negative bacterium Escherichia coli has advantages in its unmatched genetic tools and ability to act as a probiotic (5, 6). Secretion of recombinant proteins, however, is difficult in Gram-negative bacteria (7) due to their outer membrane (OM). It acts as a barrier preventing extracellular protein export via common secretory pathways, including the general secretory (Sec) pathway and the twin-arginine translocation (Tat) pathway (6), which both secrete to the periplasm. Recombinant proteins can be conjugated with a type three secretion system (T3SS) signal sequence (8) or YebF (9) for export from E. coli cells; however, these strategies release proteins as protein fusions, which may alter the structure and/or activity of the target proteins.

Gram-negative type I secretion systems (T1SSs) are unique in their ability to translocate proteinaceous cargos from the cytoplasm to the extracellular environment in one step (6, 10). While commonly described as one group, T1SSs can be subdivided into those that secrete large protein cargos (>50 kDa) and those that secrete small protein cargos (<10 kDa) (11). These two subgroups differ in their methods of processing cognate substrates and suggested models for secretion (11, 12). T1SSs are best known for export of large-protein toxins and enzymes (10, 12). A well-studied example is the Escherichia coli hemolysin A T1SS, which secretes the hemolytic toxin hemolysin A (HlyA) (10–12). The protein cargos encode a large C-terminal signal peptide (SP) that is recognized by their cognate T1SSs. Following SP recognition, proteins are extruded linearly through a membrane tunnel to the environment, where they fold for action (13). This system has been used for recombinant protein secretion, including single-chain variable-fragment antibodies (14), nanobodies (15), interferon alpha-2 (16), and lipase (16). It was discovered that the overall charge, isoelectric point, and folding rate of the cargo could each influence protein secretion efficiency (6, 17, 18). This knowledge helps us understand sequence constraints on natural protein cargos and provides insight into how these systems may be co-opted for the production and delivery of heterologous proteins (6, 14–16, 19). Although heterologous secretion via the HlyA T1SSs is successful, the long SP is retained by the exported protein, which might not be optimal for fusion protein function or delivery.

**FIG 1:**
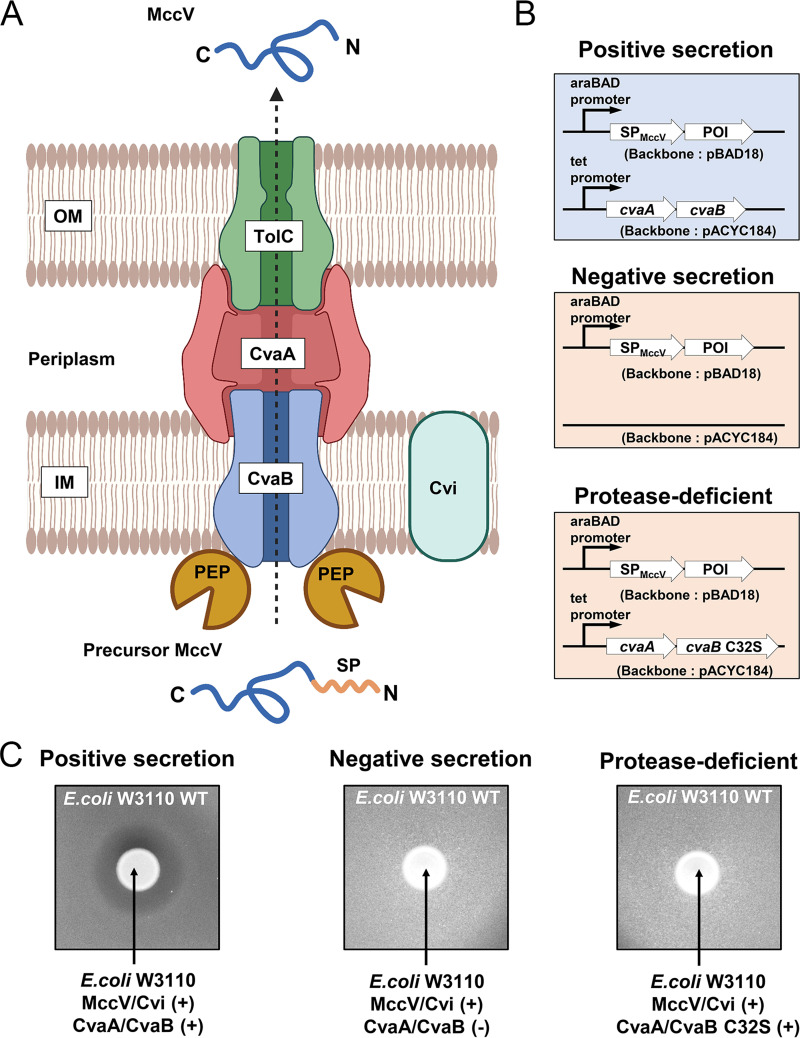
Construction of the secretion system. (A) The MccV secretion complex is composed of CvaA, CvaB, and TolC and forms a channel for movement of cargo small proteins from the cytoplasm to the extracellular environment. The 15-amino-acid signal peptide (SP) sequence of MccV (SP_MccV_) is cleaved by the peptidase domain (PEP) of CvaB during export. The N terminus and C terminus of MccV are shown as N and C, respectively. Cvi is an immunity protein that protects host bacterial cells from secreted MccV. This figure was created with BioRender.com. (B) The two-plasmid small-protein secretion system is shown. Positive-secretion strains carry a plasmid (pBAD18) expressing SP_MccV_ conjugated to the protein of interest (POI) by the *araBAD* promoter and a plasmid (pACYC184) constitutively expressing CvaA and CvaB by the *tet* (tetracycline resistance gene) promoter. Control strains encode the same POI but carry either empty pACYC184 with no CvaAB (negative secretion) or pACYC184 expressing CvaA/CvaB C32S (protease-deficient secretion). Our secretion system uses chromosomally expressed TolC or homologs. (C) The results of MccV zone-of-inhibition assays are shown. E. coli W3110 cells expressing MccV and Cvi were spotted on a lawn of susceptible E. coli W3110. Only the positive secretor generated a zone of inhibition. All samples were spotted on the sample agar plate. The results shown are representative of biological triplicates.

The less-studied subgroup of Gram-negative T1SSs specializes in the export of small-protein antibiotics (<10 kDa) called microcins (20, 21). These T1SSs are distinct in their secretion mechanism, recognizing and cleaving a short, N-terminal SP during cargo export. The most well-studied example is the microcin V (MccV, previously referred to as colicin V) T1SS. The MccV system was identified in E. coli (22–25) and is comprised of three parts: a C39 peptidase-containing ATP-binding cassette transporter (PCAT), CvaB; a membrane fusion protein (MFP), CvaA; and an outer membrane efflux protein (OMP), TolC (25) (Fig. 1A). MccV is synthesized as a 103-amino-acid precursor protein containing an N-terminal 15-amino-acid SP. The peptidase domain (PEP) of CvaB precisely cleaves the SP in an ATP-dependent manner, releasing the mature MccV for transit to the extracellular environment (26). One model for PCAT-driven small protein export is alternating-access transport (27, 28). In this model, the small-protein cargo docks into the inward-facing (cytoplasmic) central PCAT cavity. Following ATP binding, the PCAT cavity changes to face outward, which can allow bound cargo to be released across the membrane. This is different than the proposed linear-extrusion model for large-protein cargo, such as HlyA, which would require a continuous opening of the secretion channel. Thus, alternating-access transport is not generally believed to be a model for the HlyA T1SS (11, 12).

Despite their importance in bacterial competition (29–31), only 10 microcins and their cognate T1SSs have been identified among Gram-negative bacteria (20, 21). The 10 mature microcins share sequence composition similarities, including high glycine content and high hydrophobic-residue content (20). Previous reports indicate that the microcin T1SS can secrete related microcins and bacteriocins from Gram-positive bacteria with similar sequence compositions (4, 32–34), suggesting that export is not limited to the cognate cargo. However, it is unclear whether nonbacteriocin small proteins can be secreted through the MccV T1SS or how the physical and chemical features of small proteins might influence their export. This lack of information limits our understanding of the potential cargos capable of export through the MccV T1SS.

Here, we show that the MccV T1SS is a versatile small-protein secretion system capable of exporting a diverse range of synthetic and bioactive sequences. Remarkably, secretion is largely independent of the sequence’s chemical properties and only strongly limited by protein length. Furthermore, the MccV system is functional in several related species of Gram-negative bacteria adapted to gut colonization. Our work provides the first comprehensive study of cargo requirements for export through the MccV system and suggests this system’s potential as a general small-protein secretory platform in Gram-negative bacteria.

## RESULTS

### Development of a two-plasmid small-protein secretion system.

A model of the native microcin V (MccV) type I secretion system (T1SS) is illustrated in Fig. 1A. CvaB encodes a C39 peptidase domain (PEP) that cleaves the 15-amino-acid signal peptide (SP) of pre-MccV (24, 35, 36). MccV then proceeds through CvaA and TolC as it exits the cell to reach the extracellular space. An immunity protein (Cvi) is coexpressed with MccV and protects the host cell against MccV’s antibacterial action (23). In nature, CvaAB, MccV, and Cvi are encoded on the same plasmid, and TolC is expressed from the chromosome (22, 23).

To investigate small-protein features that influence export through a microcin T1SS, we first developed a two-plasmid MccV secretion system that separates the export functions from the cargo protein (Fig. 1B). The export components, CvaAB, are encoded on pACYC184 and are constitutively expressed. Small proteins designed for secretion are cloned as fusion sequences with the MccV signal peptide (SP_MccV_) on pBAD18 under arabinose-inducible control. We refer to positive secretion (PS) if the bacteria carry plasmids encoding the SP_MccV_-conjugated protein of interest (POI) and the wild-type (WT) CvaAB export system. Negative secretion (NS) refers to control bacteria that contain the same plasmids used for expression of the SP_MccV_-POI but with pACYC184 lacking CvaAB (empty vector). It was previously shown that a C32S mutation in CvaB (a change of C to S at position 32) abolished secretion of MccV due to disruption of the protease function of CvaB (26, 37). Protease-deficient secretion (PD) refers to bacteria that contain pBAD18 for expression of the SP_MccV_-POI and pACYC184 expressing CvaA with a protease-deficient CvaB mutant (C32S). Both negative- and protease-deficient secretion strains acted as controls to ensure that the observed protein secretion was due to CvaAB activity and not due to bacterial cell lysis in our experiments.

To ensure that our two-plasmid approach enabled robust cargo secretion, we first tested the expression and activity of the well-studied native MccV and its immunity protein (Cvi) in E. coli strain W3110 as a proof of concept for our approach. When plated on a lawn of sensitive E. coli cells, a zone of inhibition (ZOI) could be observed around our microcin-secreting strain, indicating that the MccV was secreted and active. We observed that only E. coli cells encoding MccV, Cvi, and WT CvaAB produced a visible ZOI against susceptible E. coli cells (Fig. 1C). Strains encoding MccV, Cvi, and empty pACYC184 or the CvaB mutant did not form a ZOI, indicating that they could not secrete MccV.

**FIG 2:**
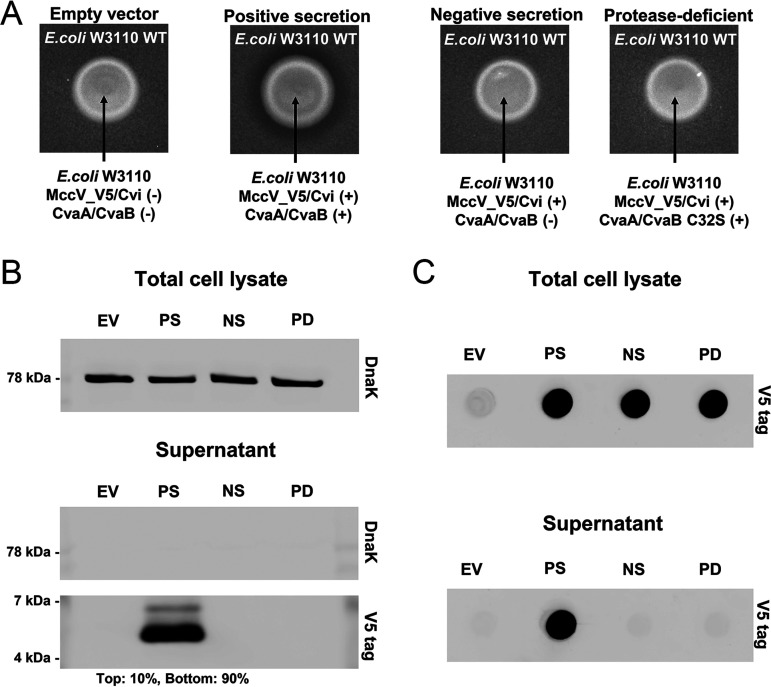
Recombinant-small-protein secretion via the MccV system. (A) Zone-of-inhibition assays were performed as described in the legend to Fig. 1C. The empty-vector (EV) strain carries empty plasmids pBAD18 and pACYC184. The positive-secretion (PS) strain encodes MccV_V5, Cvi, and CvaAB. The negative-secretion (NS) strain encodes MccV_V5 and Cvi and carries empty plasmid pACYC184. The protease-deficient secretion (PD) strain encodes MccV_V5, Cvi, and CvaA/CvaB C32S. All samples were spotted on the same agar plate. (B) Western blot detecting secreted MccV_V5 from E. coli W3110 is shown. Culture supernatant or pellet (total cell lysate) was directly suspended in sample buffer and loaded into each well. Antibody targets are named on the right side. The percentages of top band and bottom band intensity of the V5 tag from the PS supernatant sample are shown at the bottom. (C) The results of dot blotting against the V5 tag in total cell lysate and supernatant are shown. Samples from all strains (EV, PS, NS, and PD) were loaded into wells of the dot blot apparatus and detected with anti-V5 antibody. The results shown are representative of biological triplicates, and all Western or dot blot images were prepared from a single membrane.

We next generated a C-terminally V5 epitope-tagged MccV (MccV_V5) to track its secretion from E. coli W3110 by immunoblotting. Two glycine residues (GG linker) were included between MccV and the V5 tag to allow flexibility of the epitope. This MccV_V5 fusion retained its inhibitory activity (Fig. 2A). Only E. coli cells encoding MccV_V5, Cvi, and WT CvaAB could inhibit susceptible E. coli cells (Fig. 2A). This result indicates that MccV_V5 is secreted and shows the same dependency on CvaAB as the native MccV.

We induced expression for 2 h and investigated the supernatant for the presence of MccV_V5. In supernatant from our PS strain, we detected a dominant band migrating near 5 to 6 kDa (Fig. 2B), which is consistent with previous observations of MccV (24, 25, 35). We observed a weaker band migrating at a slightly higher molecular weight that likely represents the unprocessed precursor MccV_V5 (25). This suggests that a small amount of MccV_V5 (~10%) can escape without N-terminal processing under these conditions. MccV_V5 was only detected from bacteria that encoded WT CvaAB (Fig. 2B). This result is consistent with the ZOI results from the experiment whose results are shown in Fig. 2A and further supports the dependence of peptide secretion on CvaAB. The cytoplasmic protein DnaK was not observed in any of the supernatant samples but was readily observed in total cell lysate, indicating that bacteria were not lysing during the secretion of MccV_V5. To simplify the process for detecting secreted cargos, we performed dot blot analysis of MccV_V5 from supernatants and total cell lysates (Fig. 2C). While MccV_V5 was detected in all total cell lysates, it was only present in the supernatant of our PS strain, consistent with our Western blot analysis (Fig. 2B). These results support the use of our two-plasmid platform and dot blotting to detect secreted small proteins.

### Construction of a sequence-diverse synthetic-small-protein library.

The MccV secretion system has only been shown to export its native cargo and related microcins and bacteriocins, which share unusual sequence features like high glycine content and hydrophobic residue content. (4, 32, 33). Thus, it is unclear whether the MccV system can export more diverse nonmicrocin/-bacteriocin sequences and whether there are biochemical or physical limitations on protein cargo that can be secreted. To begin to examine how chemistry and length influence protein secretion through the MccV system, we generated a library of 40 random small proteins. Our library consists of four groups; each group includes 10 sequences that have the same length (10, 20, 50, or 100 amino acids) but different, randomly generated sequences. Each small protein encodes a C-terminal V5 tag, which increases the lengths of groups 1 to 4 to 26, 36, 66, and 116 amino acids, respectively (Table S1 in the supplemental material). Finally, each sequence encodes an N-terminal SP_MccV_ to direct export. We hypothesized that using small proteins of diverse composition and length would provide insight into potential chemical or physical constraints on the cargo sequences that can be exported through the MccV system. There is not a clear length distinction between a peptide and a small protein. While members of group 1 (26 amino acids) and group 2 (36 amino acids) could be referred to as peptides, members of group 3 (66 amino acids) and group 4 (116 amino acids) would not. For simplicity, we will refer to all the sequences in our libraries as being small proteins, which is the more inclusive term.

We plotted the distribution of charge and hydrophobicity for each small protein in our library (Fig. S1A) and the amino acid composition per group (Fig. S1B). For the latter, we analyzed amino acid sequences without the GG linker and V5 tag to avoid composition bias. Each of these measurements appeared well distributed across the library as a whole. Group 1 encoded the shortest, 26-amino-acid small protein and showed the narrowest distribution of charge and hydrophobicity. Based on this analysis, we deemed the proteins in our library sufficiently random in length and chemistry to begin exploring their effects on small-protein secretion.

### Protein export through the MccV system is limited by length but not composition.

**FIG 3:**
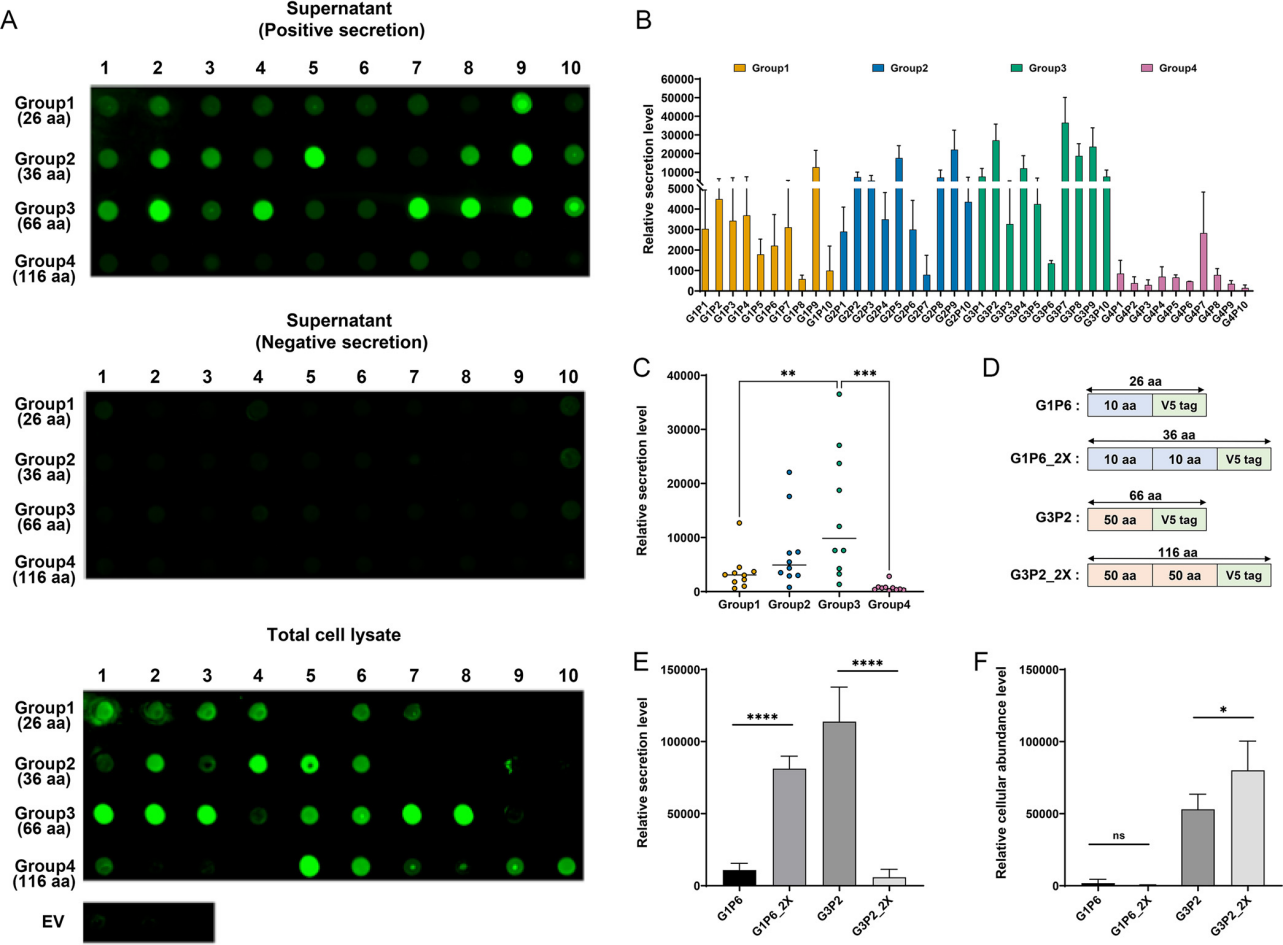
Protein secretion is dependent on sequence length. (A) The results of dot blots against the V5 tag are shown. Each small-protein group is shown on the left with the number of amino acids (aa), and the numbers on top represent each individual protein in each group. Supernatant samples include both positive and negative (no CvaAB) secretion. Total cell lysate samples were prepared by lysing negative-secretion cultures. The results for total cell lysate samples are shown along with empty-vector (EV) cell lysate samples. The results are representative of biological triplicates, and each image is from a single membrane. (B) Relative secretion levels are shown. Each group is represented by a different color, as shown above the graph. The mean values and standard deviations of biological triplicates are shown. (C) The distribution of the secretion levels (mean values from triplicates) of each group is shown. The median of the 10 secretion levels in each group is shown by a horizontal bar. Significant differences between groups are shown: **, *P* < 0.01; ***, *P* < 0.001. Adjusted *P* values were calculated by analysis of variance (ANOVA) with Tukey’s honestly significant difference (HSD) test. (D) The scheme of G1P6 and G3P2 protein 2X versions is shown. (E, F) Relative secretion and cellular abundance levels of indicated proteins are graphed. The mean values and standard deviations from six biological replicates are shown. Two-tailed *P* values from the unpaired *t* test are shown: ns, not significant (*P* > 0.05); *, *P* < 0.05; ****, *P* < 0.0001.

Each small protein in our library was expressed from E. coli W3110 with (PS) or without (NS) CvaAB to analyze its secretion level. We performed dot blotting to determine the relative amounts of each small protein in supernatants and total cell lysates after 8 h of induction (Fig. 3A). To calculate secretion levels, each signal was first normalized by the cell density (optical density at 600 nm [OD_600_]) of its respective culture to account for possible differences in bacterial abundance. Then, we subtracted the NS signal intensity to account for the background signal. The differential signal intensity, or relative secretion level, of each small protein is graphed in Fig. 3B, and the distribution of secretion levels is shown in Fig. 3C.

Remarkably, and despite their having little sequence relationship to the native cargo, all sequences in groups 1 to 3 (26 to 66 amino acids) were secreted above the background level, and in general, the secretion levels increased from groups 1 to 3 (Fig. 3B and C). Interestingly, the secretion levels abruptly dropped in group 4 (116 amino acids) as a whole (Fig. 3A and B). This decrease was not due to lower cellular abundance of group 4 sequences, since the signals from total cell lysates were similar across all groups (Fig. 3A and Fig. S2A). We hypothesize that the MccV system has a preference for a cargo length between its native substrate size (MccV = 88 amino acids) and the 116-amino-acid length of group 4 sequences.

If our hypothesis was correct, we expected that a group 1 sequence (26 amino acids) would have a higher secretion level if its length was increased to the group 2 length (36 amino acids) and a group 3 sequence (66 amino acids) would have a lower secretion level if its length was increased to the group 4 length (116 amino acids). To test this concept, we selected two sequences: G1P6 (group 1 small protein 6) and G3P2 (group 3 small protein 2). We generated new proteins, G1P6_2X (36 amino acids) and G3P2_2X (116 amino acids), whose sequences were lengthened by adding a direct repeat of their respective amino acid sequence (maintaining a single V5 tag) and maintained biochemical properties similar to those of the parent proteins (Fig. 3D). We measured their relative secretion and cellular abundance levels by dot blotting (Fig. 3E and F). G1P6_2X had a significantly higher secreted level than the parent sequence, G1P6 (Fig. 3E). The opposite result was observed when comparing secretion of G3P2 and G3P2_2X. In this case, the longer G3P2_2X protein showed significantly less secretion than the shorter parent sequence, G3P2 (Fig. 3E). In both cases, changes in secretion levels did not correlate with similar changes in cellular abundances (Fig. 3F). These results support our hypothesis that protein length is the dominant factor influencing cargo export through the MccV T1SS.

### Protein export is moderately affected by additional sequence characteristics.

In addition to length, we questioned whether other properties influenced protein export through the MccV T1SS. We first analyzed the global chemical characteristics of all group 1 to 3 sequences (Fig. S2). We excluded group 4, since their generally poor export was most likely driven by their length. We did not observe a correlation between the protein secretion level and total protein charge (*r* = −0.03469, *P > *0.05) (Fig. S2B) or charge magnitude (*r* = −1.033, *P > *0.05) (Fig. S2C), but we did observe a moderate negative relationship (*r* = −0.4029, *P < *0.05) between hydrophobicity and secretion level (Fig. S2D). However, none of these global features provided a strong correlate. We next tested whether codon usage impacted the secretion of small proteins. To estimate, we used the codon adaptation index (CAI) of each small protein (38, 39). A moderate positive relationship was observed between CAI and secretion level (*r* = 0.4430, *P < *0.05) (Fig. S2E). Similarly, we observed a moderate positive relationship (*r* = 0.5775, *P < *0.001) (Fig. S2F) between cellular abundance and secretion level. We also considered that a protein’s intracellular stability might influence its secretion. More-stable proteins may have more time to productively interact with the MccV T1SS. To investigate this possibility, we selected proteins G3P2 (group 3 small protein 2) and G3P3 (group 3 small protein 3). These proteins had similar cellular abundances, but G3P2 had a significantly higher secretion level (Fig. 3 and Fig. S2G). We used pulse-chase protein labeling followed by immunoprecipitation to track intracellular protein levels over time. We found that the relative degrees of stability of G3P2 and G3P3 were indistinguishable over time (Fig. S2H and I). This result suggests that protein stability is unlikely to be a general driver of secretion levels.

Our results indicate that, other than length (Fig. 3), there does not appear to be a set of sequence attributes that can broadly explain or predict the variations in protein secretion through the MccV T1SS. The lack of correlation between yield and sequence information is observed for all other protein secretion systems from bacteria as well (6, 40, 41). Nevertheless, the MccV T1SS appears capable of exporting a broad diversity of small-protein sequences, which provides many opportunities for small-protein investigation.

### The MccV system can export heterologous small proteins as efficiently as the native cargo.

**FIG 4:**
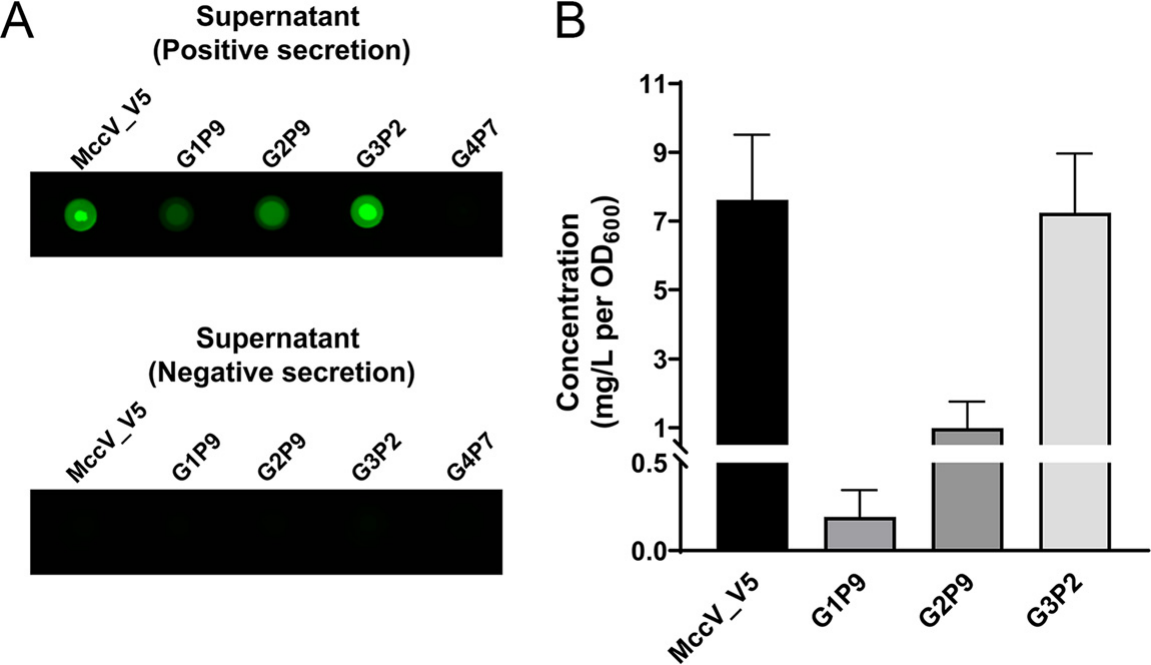
Secretion capacity of the MccV system. (A) Dot blot results showing V5 tag signal intensities of positive- and negative-secretion supernatants of strains expressing selected small proteins (MccV_V5, G1P9, G2P9, G3P2, and G4P7) are shown. A representative dot blot image prepared from a single membrane is shown. (B) The concentrations (mg/L per OD_600_) of the selected proteins are shown as a graph. The mean values and standard deviations from at least four biological replicates are shown.

A diverse set of protein sequences can be exported through the MccV T1SS (Fig. 3). We next asked how well heterologous proteins were exported compared to the native cargo MccV in terms of yield. To investigate this question, we quantified the amount of V5-tagged natural substrate (MccV_V5) and a well-secreted protein (Fig. 3B) from each of our four library groups. Supernatant from E. coli cells secreting MccV_V5, G1P9, G2P9, G3P2, and G4P7 was dot blotted after 8 h of induction (Fig. 4A). The secretion level was converted to mg/L per OD_600_ based on a standard curve generated using a commercially synthesized V5-tagged small protein (Fig. S3). The G1P9, G2P9, and G3P2 supernatant concentrations were in the high μg/L to low mg/L (0.19 to 7.25 mg/L) per OD_600_ range (Fig. 4B). This range is similar to the previously reported purification yield of MccV secreted via its cognate system (0.5 mg to 4 mg/L, OD_600_ is not reported) (35). Interestingly, the concentrations of G3P2 and MccV_V5 were similar, suggesting that a heterologous protein can be secreted at a level comparable to the native cargo. As anticipated, the 116-amino-acid long G4P7 protein secretion level was low (Fig. 4A) and out of range of our standard curve.

### Abundance of secreted small proteins is influenced by host strain and temperature.

Given the potential uses of the MccV T1SS in small-protein production, we questioned whether export could be further optimized. All our tests so far had been conducted in E. coli K-12 strain W3110 at 37°C. In the experiment whose results are shown in Fig. 3, we observed that G1P5, G1P10, G2P6, G2P7, G3P3, and G3P5 had relatively lower secretion levels. We hypothesized that using a bacterial strain designed for protein production through deletion of cellular proteases [E. coli strain BL21(DE3)] and lowering the growth temperature, which are general strategies for heterologous protein expression optimization (42), could increase secretion levels. To test this hypothesis, the secretion levels from E. coli W3110 grown at 30°C, E. coli BL21(DE3) grown at 37°C, and E. coli BL21(DE3) grown at 30°C were measured and normalized to the levels produced under the conditions used in the experiment whose results are shown in Fig. 3 (E. coli W3110 at 37°C) (Fig. S4). Lowering the growth temperature and/or using BL21 cells significantly increased the secretion levels of 5 of the 6 proteins (G1P5, G2P6, G2P7, G3P3, and G3P5), from 2- to 17-fold. This might be due to a lowered translation rate at the lower temperature (43) as, theoretically, ribosome stacking or collision, which cause degradation of a translating protein, would be avoided at a lower translation rate (44). Only the expression of G1P10 was not significantly increased. Lowering the induction temperature reduces growth and protein translation rates, which can aid proper folding of foreign sequences (43, 45). Thus, the improved folding of secretion system components or reduced cargo protein aggregation might increase the amount of secretion at lower temperatures. The use of E. coli BL21(DE3) might increase protein secretion levels due to deficiency of Lon and OmpT proteases, as Lon degrades many heterologous proteins (46) and the outer-membrane protease OmpT degrades extracellular proteins (47). No single change (temperature or strain) consistently increased secretion. These results indicate that secretion of proteins through the MccV T1SS can be strongly influenced by environmental conditions and host strain, but the impact varies by cargo sequence.

### Secretion of bioactive small proteins by the MccV system.

Studies of non-native-cargo export via the MccV system have focused on microcins and bacteriocins, which share similar sequence compositions (4, 32–34). We have shown that a range of random sequences can also be exported. To expand our knowledge, we sought to test the secretion of bioactive small proteins from various organisms. We selected four examples within the length constraints defined above that have diverse biological functions: pediocin PA-1, α-factor, eglin C, and epidermal growth factor (EGF). The functions and properties of the small proteins are listed in Table S2. Each sequence was fused with the N-terminal SP_MccV_ to direct its export.

**FIG 5:**
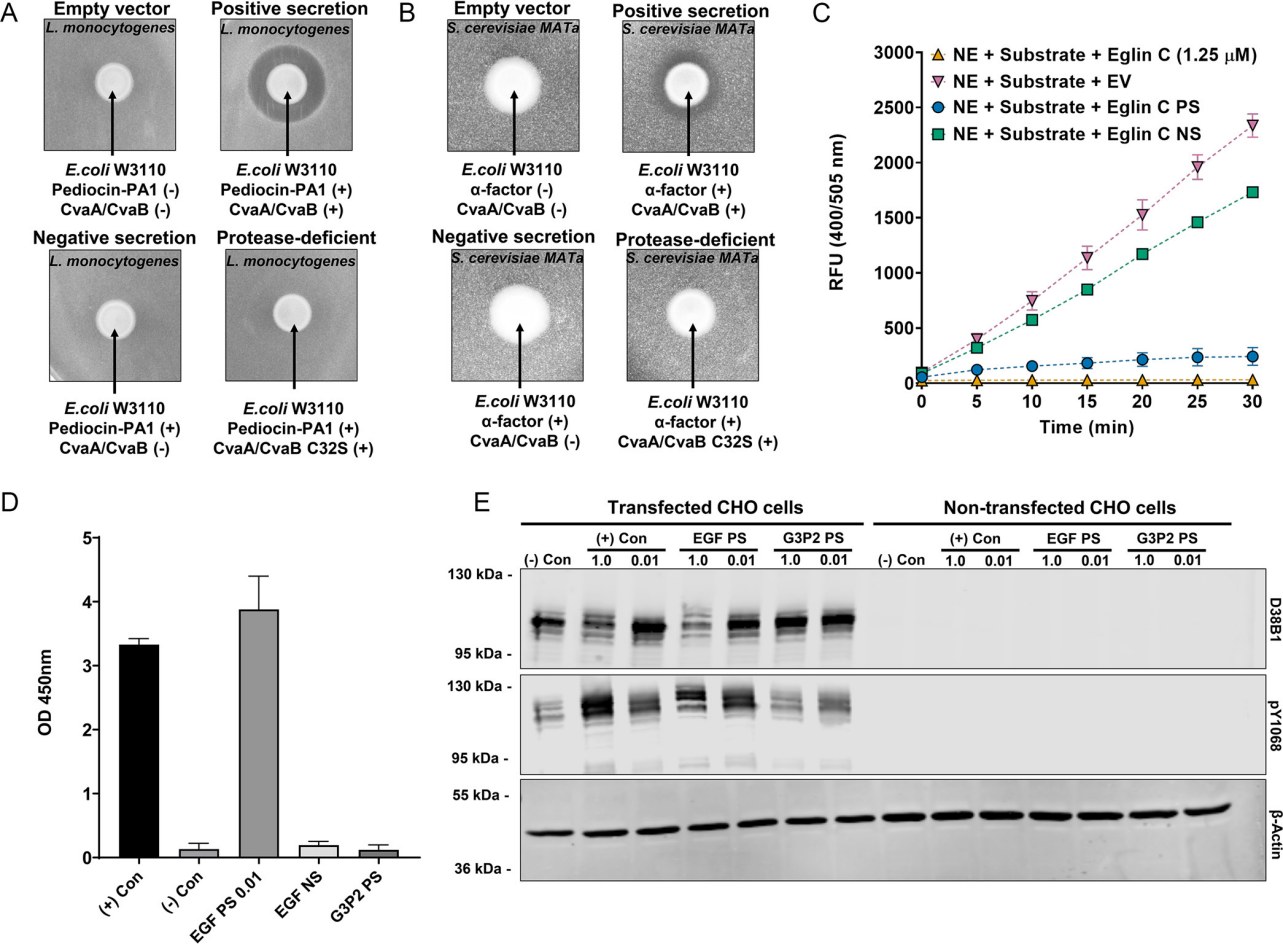
Bioactive proteins are secreted via the MccV system. (A and B) The results of two zone-of-inhibition assays are shown. The images shown are representative of biological triplicates. (C) The results of neutrophil elastase (NE) activity assays are shown. Relative fluorescence levels of samples at each time point are shown. RFU, relative fluorescence units. All samples contained NE and substrate, and 1.25 μM recombinant eglin C, empty vector (EV) supernatant, eglin C positive-secretion (PS) supernatant, or eglin C negative-secretion (NS) supernatant was separately added into each sample. The mean values and standard deviations of biological triplicates are plotted. (D) The results of ELISA for human epidermal growth factor (EGF) are shown. OD_450_ values indicate relative amounts of human EGF in samples. (+) Con (positive control) represents a sample containing standard EGF (1 ng/mL), and (−) Con (negative control) represents a sample containing assay buffer. EGF PS 0.01 represents 100-fold-diluted EGF positive-secretion supernatant in fresh medium. EGF NS represents EGF negative-secretion supernatant (no CvaAB). G3P2 PS represents G3P2 positive-secretion supernatant. The mean values and standard deviations from biological duplicates are shown. (E) The results of Western blot assays are shown. Human EGF receptor (EGFR)-transfected and nontransfected CHO cells were treated with each sample: (−) Con, untreated; (+) Con, standard EGF (100 ng/mL); EGF PS, EGF positive-secretion supernatant; and G3P2 PS, G3P2 positive-secretion supernatant (nonspecific protein control). Cells were treated with either undiluted sample (1.0) or 100-fold-diluted sample (0.01) in fresh medium, and cell lysates were subjected to Western blotting against EGFR (D38B1), phosphorylated EGFR (pY1068), and β-actin. The blots are representative images of biological duplicates.

The MccV system was previously shown to be able to secrete Gram-positive bacteriocins (4, 32, 33). We began our study by extending this observation and testing the secretion of pediocin PA-1, a bacteriocin natively produced by the Gram-positive bacterium Pediococcus acidilactici and active against the Gram-positive bacterium Listeria monocytogenes (48). As we did for MccV (Fig. 1C), we performed ZOI assays by secreting pediocin PA-1 from E. coli W3110 spotted on a lawn of L. monocytogenes. When E. coli expressed both pediocin PA-1 and CvaAB, it inhibited L. monocytogenes growth and produced a ZOI (Fig. 5A). No ZOI was observed with our NS strain (no CvaAB) or the PD strain (CvaB C32S mutant), confirming the CvaAB-dependent secretion and action of pediocin PA-1. This result is consistent with previous findings (4, 32, 33) that showed that the MccV system is capable of secreting nonnative small bacteriocins.

We continued our study by progressing evolutionally outward with secreted cargo. We used the ZOI test to assay α-factor production. α-Factor is a pheromone released by Saccharomyces cerevisiae mating-type alpha cells that activates the G protein-coupled receptor (GPCR) Ste2p, causing cell cycle arrest in susceptible S. cerevisiae strains (*MAT***a**) (49). The results in Fig. 5B show that, when E. coli W3110 expressed both α-factor and CvaAB, it inhibited the growth of susceptible S. cerevisiae *MAT***a** cells. This indicates that the MccV system can secrete proteins active against GPCRs and impact evolutionarily distant organisms.

We next tested whether the MccV system would secrete a metabolic inhibitor. Eglin C is a naturally occurring small protein from leeches and a potent inhibitor of serine proteases, including elastase (50). Eglin C has been investigated for the treatment of gastrointestinal conditions (51). We tested the ability of secreted eglin C to inhibit neutrophil elastase (NE) compared to commercial eglin C. The assay measures the cleavage of a substrate-fluorophore conjugate by NE; hydrolysis by NE releases the fluorescent group, which can then be detected. As shown by the results in Fig. 5C, NE treated with empty-vector E. coli W3110 supernatant successfully degraded the substrate, and fluorescence increased over time, indicating that NE was active. In contrast, NE activity was strongly inhibited by the addition of commercial eglin C or eglin C PS supernatant. The fluorescence signal from NE treated with eglin C NS supernatant increased over time, indicating that NE remained active. However, the rate was slightly lower than that of the empty-vector NE control, suggesting that a small amount of eglin C was present in the NS supernatant. This may be due to a small amount of eglin C released by cell lysis and detected by this sensitive assay. Overall, this result indicates that eglin C is successfully secreted via the MccV system and retains its activity.

Finally, we tested a more complex small protein, human epidermal growth factor (EGF), that stimulates the growth of epithelial cells through binding to a specific tyrosine kinase receptor (52). Mature EGF has three disulfide bonds. However, EGF is commonly purified from bacteria and refolded, suggesting that the primary sequence is sufficient to develop the mature fold under oxidative conditions (53). Prior to testing the bioactivity of EGF, we measured the relative amount of EGF in supernatant from E. coli W3110 grown in mammalian cell culture medium (Fig. 5D) by colorimetric enzyme-linked immunosorbent assay (ELISA). In addition to the EGF NS control, we also included PS supernatant containing the random small protein G3P2 as a nonspecific control. We had to dilute the EGF PS supernatant 100-fold to obtain an OD_450_ value similar to that obtained with 1 ng/mL of the EGF standard, suggesting that our supernatant contained ~100 ng/mL EGF. No signal was detected from EGF NS supernatant or G3P2 PS supernatant. We then assayed the ability of E. coli-secreted EGF to activate the EGF receptor (EGFR) in cell culture. Transfected CHO cells expressing hemagglutinin (HA)-tagged EGFR were treated with purified EGF standard, EGF PS supernatant, or G3P2 PS supernatant. Cell lysates were immunoblotted for the presence of EGFR, phosphorylated (activated) EGFR, and β-actin (loading control) (Fig. 5E). EGF standard and EGF PS supernatant increased EGFR phosphorylation to similar levels, while no change in EGFR phosphorylation was observed from the G3P2 PS (nonspecific protein). No signal for EGFR was observed in nontransfected CHO cells. These results indicate that EGF can be secreted by E. coli through the MccV system, obtain its active form, and activate its target human receptor.

To further characterize these secreted proteins, we purified eglin C and EGF for analysis, since both are derived from nonmicrobial sources and thus represent sequences far removed from native bacterial secretion. Each protein was constructed with a C-terminal Strep-tag and secreted into 0.5 L of bacterial growth medium. Proteins were purified from the supernatant via affinity chromatography, and purity was assessed by SDS-PAGE (Fig. S5A). We observed a single band for each protein near the expected molecular weight. When analyzed by mass spectrometry, a single, dominant signal was observed for each protein (EGF_strep, 7,370.34 Da, and eglin C_strep, 9,244.74 Da) (Fig. S5B and C). Each signal matched the theoretical mass of the respective protein after SP_MccV_ cleavage within <1 Da. For EGF, this profile matched the mature disulfide bond-containing form. Minor mass signals were observed, but none matched the mass of the proteins containing additional SP_MccV_ sequence residues. This indicated that SP_MccV_-cleaved proteins were the dominant species in the supernatant. The yields were 0.57 mg/L (EGF_strep) and 1.11 mg/L (eglin C_strep) per OD_600_ under these test conditions. These yields are consistent with the range for the MccV system (0.19 to 7.25 mg/L per OD_600_) that we estimated by dot blotting (Fig. 4B).

### The MccV system is functional in related Gram-negative bacteria.

**FIG 6:**
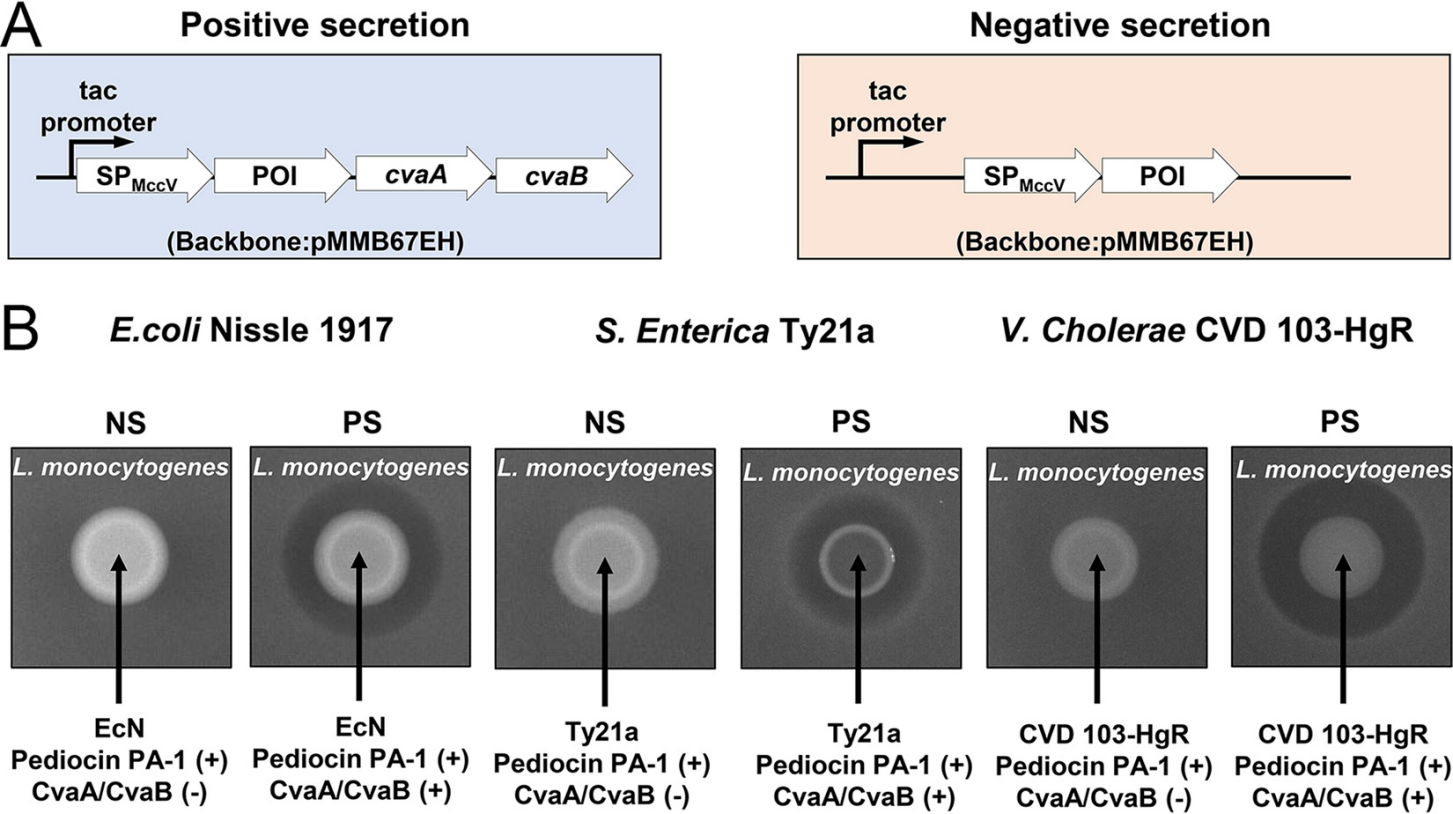
Compatibility of the MccV system with other Gram-negative bacteria. (A) The one-plasmid small-protein secretion system is shown. A protein of interest (POI) conjugated with the MccV signal peptide (SP_MccV_) is expressed with CvaAB (positive secretion) or without CvaAB (negative secretion). (B) The results of pediocin PA-1 zone-of-inhibition assays are shown. Cultures of bacteria containing either the pediocin PA-1 negative-secretion (NS) or positive-secretion (PS) system were spotted on a lawn of L. monocytogenes and 1 mM IPTG. The per-species pair of NS and PS was spotted on the same agar plate and the spot images were prepared from the same plate picture, but separate plates were used for different bacterial species. The results shown are representative of biological triplicates.

We have shown that the MccV system can secrete heterologous small proteins from E. coli strains W3110 and BL21(DE3). Microcin-exporting T1SSs are predicted to be broadly distributed across Gram-negative bacteria (54), suggesting that the MccV system may retain its function in non-E. coli strains. To make the secretion system more amenable to a wider range of bacteria, we cloned it into the broad-host-range plasmid, pMMB67EH (Fig. 6A). This configuration allows the expression of the protein of interest and CvaAB under IPTG-inducible control. For testing purposes, we used pediocin PA-1 as the cargo to provide a simple ZOI readout. We transformed this plasmid and the negative-control plasmid lacking CvaAB into E. coli Nissle 1917, Salmonella enterica Ty21a, and Vibrio cholerae CVD103-HgR. E. coli Nissle is a commonly used probiotic strain (5). S. enterica Ty21a and V. cholerae CVD103-HgR have been explored as vaccine strains of human pathogens and are shown to be safe for human use (55, 56). Each bacterial strain expressing SP_MccV_-pediocin and CvaAB was able to inhibit the growth of L. monocytogenes (Fig. 6B), indicating that the MccV system is functional in a probiotic E. coli strain and at least two additional Gram-negative species capable of gut colonization.

## DISCUSSION

Previous studies of heterologous small-protein secretion via MccV systems focused on the production of microcins and bacteriocins, which share similar sequence compositions (4, 32–34). Remarkably, we found that the MccV T1SS could secrete a much wider range of synthetic and bioactive small proteins with diverse sequence contents. Despite the high glycine and hydrophobic-residue contents of native microcins, we found no strong association between small-protein sequence content or chemistry and their secreted abundances. The only constraint on secretion through the MccV T1SS that we could identify was sequence length. The limited ability of the MccV T1SS to export 116-amino-acid-long sequences (group 4 sequences and G3P2_2X) may be explained by recent observations of the structure of a related PCAT, PCAT1, from the Gram-positive bacterium, Clostridium thermocellum (27, 28). An alternating-access model was proposed for its substrate transport through PCAT1. Once the signal peptide (SP) of the substrate is recognized by the flexible peptidase domain (PEP) of PCAT1, the translocating substrate inserts via its C-terminal region into the side of central cavity of the PCAT1 transmembrane domain. This cavity faces inward in the absence of ATP binding. Following ATP binding, PCAT1 changes from the inward- to the outward-facing-cavity form. This enables the cargo in the PCAT1 transmembrane conduit that is captured by the PEP to be released into the extracellular space following cleavage of its SP. As a member of the PCAT family, CvaB may also follow this model. Substrates that are too long may not be efficiently loaded into the transmembrane conduit of CvaB or may hamper the conformational change to the outward form to translocate the substrate through the next parts of the secretion complex, CvaA and TolC. Another hypothesis is about TolC protein recruitment by the substrate. It is not clearly elucidated in the MccV T1SS, but in the hemolysin A (HlyA) T1SS, HlyA recruits TolC to assemble the HlyB (ABC transporter)-HlyD (membrane fusion protein)-TolC complex for its secretion (57). Considering that PhoA conjugated with the MccV SP was translocated to the inner-membrane-facing periplasm (23), it may be possible that a cargo that is too long cannot recruit TolC properly and remains stuck in the inner-membrane–periplasm region. However, G4P7 (group 4 small protein 7) did show a modest level of secretion, indicating that length may not be a strict limitation.

The promiscuity of the MccV system opens several opportunities for small protein research and delivery. We show that, in addition to bacteriocins like pediocin PA-1, the MccV system can secrete a range of nonbacterial bioactive small proteins, as exemplified through our secretion of a yeast pheromone (α-factor), a protease inhibitor (eglin C), and a human hormone (epidermal growth factor). A key benefit of the MccV system is the ease of use. Simply appending the MccV SP to the cargo sequence appears sufficient to drive the export of diverse small proteins. As synthesis of small proteins remains chemically challenging (7, 58), the MccV system could provide a facile method for small-scale protein production. An important consideration for this use is the required yield. Over 8 h, E. coli W3110 secretes 0.19 to 7.25 mg/L of small protein per OD_600_. This yield is comparable to those of other Gram-negative secretion systems, such as the HlyA T1SS (nanobodies, 0.3 to 1.3 mg/L) (15) or flagellar T3SS (cutinase, 0.16 mg/L, and human growth hormone, 0.15 mg/L) (59). Another strategy (60) can produce higher small-protein yields under fermentation (teriparatide, >2 g/L) but requires cumbersome cell lysis, inclusion body recovery, and fusion protein cleavage to recover the desired protein. We have not intensively attempted to optimize export, but our studies using E. coli BL21(DE3) lacking proteases and lowering the growth temperature indicate that protein secretion abundance can be improved (Fig. S4). Investigations of several protein export systems have shown that secretion efficiency is highly dependent on the cargo, and solid rules for optimal secretion for any system remain elusive (6, 40, 41). Ideal conditions for small-protein secretion through the MccV system likely have similar sequence dependency. In its current form, the MccV system may provide an opportunity for rapid small-scale analysis of novel small proteins or libraries. The protease domain of CvaB makes a precise cut at the end of the N-terminal signal peptide (SP) sequence, releasing the mature protein into the environment without additional residual amino acids, unlike other systems, where the final product retains the sequences required for export (8, 9, 61); this may prove to be an advantage for small-protein production.

As highlighted by investigations of other secretion systems (62–65), extracellular secretion via the MccV system could provide opportunities for *in vivo* small-protein delivery. Oral delivery of proteins is challenging because they are quickly degraded during passage through the stomach (66). Many bacteria can survive this passage, so they could be used to overcome this barrier and begin delivering bioactive small proteins once in the gut to affect a wide range of outcomes, such as altering metabolism, influencing immune responses, or treating cancers. The MccV system is active in several bacteria that have tropisms for colonizing different gut locations, which could be leveraged to deliver small proteins to specific areas of the intestinal tract and act as on-site production factories to generate a constant localized flow of small proteins for sustained effects. The use of bacteria like Salmonella may even offer the chance for intracellular delivery. Bacteria that colonize other areas of our body could be used similarly for niche-specific delivery.

## MATERIALS AND METHODS

### Bacterial and yeast strains, growth conditions, and genetic modification.

The bacterial and yeast strains used in this study are listed in Table S3. For plasmid construction and molecular cloning, E. coli DH5α competent cells (catalog number C2987H; New England Biolabs) were used. All Gram-negative bacteria were grown in lysogeny broth (LB) at 37°C with shaking at 220 rpm, unless otherwise stated. L. monocytogenes was grown in tryptic soy broth (TSB) at 37°C with shaking at 220 rpm. S. cerevisiae was grown in yeast-peptone-dextrose (YPD) medium at 30°C with shaking at 275 rpm. As required, the following antibiotics were used: carbenicillin at 75 μg/mL, kanamycin at 50 μg/mL, streptomycin at 100 μg/mL, and chloramphenicol at 10 μg/mL.

### Construction of plasmids.

Standard techniques in molecular cloning were used to construct plasmids (67). All plasmids, primers, and gBlocks used in this study are listed in Tables S4 and S5. Primers and gBlocks were ordered from Integrated DNA Technologies (IDT). Random synthetic small-protein inserts for cloning were built by using either reduced random-codon (NNK)-containing primer sets or gBlocks and cognate primer sets. The gBlocks contain nucleotide sequences that are reverse translated with codon optimization from randomly generated small protein sequences (https://www.genscript.com/sms2/index.html). Other inserts containing small proteins of interest were constructed by using either codon-optimized open reading frame (ORF)-containing gBlocks or primers, cognate primer sets, and the selected plasmids mentioned below.

For the two-plasmid secretion system, plasmid pBAD18 or its derivative pSK00, which encoded the MccV SP, were used to express small proteins of interest. Each small protein of interest was conjugated with the MccV signal peptide (CvaC15, MRTLTLNELDSVSGG) at the N terminus and cloned into pBAD18. If required, the V5 tag with two additional N-terminal glycine residues (GGGKPIPNPLLGLDST) was conjugated at the C terminus. We used pHK22 (22) as a template to amplify the *cvaA* and *cvaB* genes. CvaAB were cloned into pACYC184 for constitutive expression. We refer to this plasmid as pSK01. We also constructed pSK02 by introducing a point mutation in *cvaB* of pSK01 to express a mutant-type CvaB (C32S).

For the one-plasmid secretion system, the broad-host-range vector pMMB67EH was used to construct pSK03. Amplified *cvaA* and *cvaB* as described above were cloned into pMMB67EH. Pediocin PA-1 was cloned into pSK03 using primers and gBlocks described in Table S4.

### ZOI assay.

To observe the zone of inhibition (ZOI) caused by MccV or MccV_V5, E. coli K-12 W3110 wild type was used as the susceptible strain. An overnight culture of the susceptible strain was diluted to an OD_600_ of 0.001 in LB agar medium with 0.2% (vol/vol) arabinose and solidified. Overnight cultures of the secreting strains, including empty-vector, positive-secretion, negative-secretion, and protease-deficient-secretion (Fig. 1) strains, were centrifuged at 5,000 × *g* for 5 min, and the pellets were resuspended in 100 μL of fresh LB medium. Amounts of 5 μL of the suspensions were spotted on the solidified agar, and pictures were taken after overnight incubation at 37°C.

Similarly, L. monocytogenes was used as the susceptible strain to detect the ZOI caused by pediocin PA-1. An overnight L. monocytogenes culture was diluted to an OD_600_ of 0.001 in TSB agar with 0.2% (vol/vol) arabinose (Fig. 6B) or 1 mM IPTG (isopropyl β-d-thiogalactopyranoside) (Fig. 6B) and solidified. The overnight cultures of E. coli W3110, including empty-vector, positive-secretion, negative-secretion, and protease-deficient-secretion strains (Fig. 6B), and E. coli Nissle, S. enterica, and V. cholerae containing either positive- or negative-secretion systems (Fig. 6B) were enriched and spotted in the same way as described above. Pictures were taken after overnight incubation at 37°C.

S. cerevisiae CMY 740-1D was used as the susceptible strain to detect ZOI caused by secreted α-factor. An overnight culture was diluted to an OD_600_ of 0.01 in yeast-peptone-glycerol (YPG) agar medium with 0.2% (wt/vol) arabinose and solidified. Overnight cultures of secreting strains (E. coli W3110) were enriched as described above, and 5-μL amounts were spotted on the solidified YPG agar plate. Pictures were taken after 30 h of incubation at 30°C.

### Western blotting.

To detect MccV_V5 (Fig. 2), an overnight E. coli W3110 liquid culture was diluted in LB medium to an OD_600_ of 0.5. The culture was induced with 0.2% (vol/vol) arabinose for 2 h and normalized to an OD_600_ of 1.0. A 250-μL amount of culture was taken and centrifuged at 5,000 × *g* for 5 min to separate the supernatant and cell pellet. The Invitrogen Novex Tricine gel system (Thermo Fisher Scientific) was used to perform SDS-PAGE. The supernatant and cell pellet were each suspended in Tricine SDS sample buffer (catalog number LC1676) with sample reducing agent (catalog number NP0009), and 10 μL was loaded into each well of 16% Tricine gel (catalog number EC66952) with 10 μL of SeeBlue plus2 pre-stained protein standard (catalog number LC5925) and migrated with Tricine SDS running buffer (catalog number LC1675). Then, the small proteins were transferred to a nitrocellulose membrane (catalog number LC2000). The membrane was blocked with 5% (wt/vol) low-fat milk in TTBS (50 mM Tris-HCl, pH 7.5, 150 mM NaCl, 0.05% Tween 20 [vol/vol]), and proteins were labeled by incubation with the selected primary antibody, either anti-V5 antibody (catalog number V8012; Sigma-Aldrich) or anti-DnaK antibody (catalog number ADI-SPA-880; Enzo), diluted 1:5,000 in 1% bovine serum albumin (BSA) in TTBS. Li-Cor IRDye 800CW goat anti-mouse IgG (catalog number 926-32210; Li-Cor Biosciences) was used as the secondary antibody, diluted 1:5,000 in 5% (wt/vol) low-fat milk in TTBS. The Li-Cor Odyssey Clx near-IR imaging system was used for visualizing. Band intensities were measured using Image Studio software (https://www.licor.com/bio/image-studio-lite/).

### Dot blotting.

E. coli culture samples for dot blotting were grown either using test tubes or in a 96-well deep-well plate (catalog number 503501; NEST Scientific). When using a deep-well plate, a single colony was inoculated into 1 mL of LB medium in each well and the plate incubated at 37°C with shaking at 1,000 rpm. The plate was sealed with a permeable membrane (catalog number BEM-1; Diversified Biotech) for proper air circulation. After overnight growth, cultures were diluted in 500 μL of fresh LB medium to an OD_600_ of 0.5 with 0.2% (wt/vol) arabinose and incubated at 37°C with shaking at 1,000 rpm for 8 h. The plate was centrifuged at 4,000 rpm for 10 min to collect supernatant samples. A nitrocellulose membrane (catalog number 10600010; GE Healthcare Life Sciences) was inserted into a 96-well Bio-Dot microfiltration apparatus (catalog number 1706545; Bio-Rad), and 100 μL of each supernatant was loaded onto a well in the apparatus and filtered following the manufacturer’s protocol. To perform dot blotting for total cell lysate samples, induced cultures were boiled for 20 min and centrifuged at 5,000 × *g* for 5 min to separate supernatant and cell debris. Amounts of 100 μL of the collected supernatants were loaded onto wells. After the samples passed through the membrane, the membrane was washed twice with TTBS and removed from the apparatus. Blocking, antibody incubation, visualization, and signal intensity calculations were done as described above in “Western blotting.” The raw dot blot signal intensity of the supernatant sample was divided by the OD_600_ value of the original culture. Then, the normalized intensity of negative secretion (no CvaAB) was subtracted from the normalized positive-secretion intensity. The values obtained are referred to as “relative secretion level” in Fig. 3 and Fig. S2. The raw dot blot signal intensity of the total cell lysate sample was divided by the OD_600_ value of the culture. Next, the normalized intensity of the total cell lysate was subtracted from the intensity of the empty vector culture. The values obtained are referred to as “relative cellular abundance level” in Fig. 3 and Fig. S2. We confirmed that cell lysate did not influence the linearity of the dot blot signal. In the distribution of relative cellular abundances and the correlation assay for relative secretion levels versus relative cellular abundance levels (Fig. S2A and F), we considered the cellular abundance of seven small proteins (G1P5, G1P10, G2P7, G2P8, G2P9, G2P10, and G4P3) to be zero, since the calculated cellular abundance levels of the proteins were less than zero.

### Secreted-small-protein quantification.

Positive- and negative-secretion supernatants of G1P9, G2P9, G3P2, and G4P7 (Fig. 5) were diluted in fresh LB, and 100-μL amounts of the diluents were dot blotted. Supernatant samples were diluted accordingly to be in the range of the standard curve. Signal intensity was normalized as described above. Secreted-small-protein concentrations were determined by comparison to a standard curve (signal intensity versus micromole). The standard small protein (SFRNGVGSGAKKTSFRRAKQGGKPIPNPLLGLDST) was synthesized by GenScript with ≥90% purity. The secreted-small-protein concentrations were converted to mg/L per OD_600_.

### Elastase inhibition assay.

Eglin C positive-secretion, negative-secretion, and empty-vector-containing E. coli W3110 strains were grown overnight and 100-fold diluted in fresh LB medium. Once the cultures reached an OD_600_ of 0.5, the medium was replaced with M9 minimal medium supplemented with 0.4% (vol/vol) glycerol and 0.2% (wt/vol) arabinose and grown overnight for induction. Cell-free supernatants were collected by centrifuging the cultures at 5,000 × *g* for 5 min and filtering them using a polyethersulfone (PES) 0.22-μm filter membrane (catalog number 229747; Celltreat Scientific). The protease inhibition activities of the samples obtained were tested using a neutrophil elastase inhibitor screening kit (catalog number ab118971; Abcam) following the manufacturer’s protocol. For a positive control, *N*-acetyl-eglin C was purchased from Enzo Life Sciences (catalog number ALX-201-006-MC01) and diluted at 1.25 μM in empty-vector supernatant prepared as described above.

### ELISA.

EGF positive-secretion, negative-secretion, and G3P2 positive-secretion E. coli W3110 strains were grown overnight and diluted 100-fold in fresh LB medium. Once the cultures reached an OD_600_ of 0.5, the medium was replaced with Ham’s F-12 medium (catalog number 11765070; Thermo Fisher Scientific) supplemented with 1 mg/mL BSA and 0.2% (wt/vol) arabinose and the cultures grown overnight for induction. Cell-free supernatants were collected as described above, and the supernatants were directly used to detect secreted EGF by using a human EGF ELISA kit (catalog number EK0325; Boster Bio), following the manufacturer’s protocol. Standard EGF provided by the company was used as a positive control.

### Mammalian cell culture, transfection, and EGFR phosphorylation assay.

CHO-K1 cells (catalog number CCL-61; ATCC) were grown in six-well plates at 1 × 10^6^ cells/well and transfected with 1.5 μg of pcDNA-EGFR-HAtag DNA using polyethyleneimine (PEI) (catalog number NC1014320; Fisher Scientific) as described previously (68) at a ratio of 3:1. After 18 h, cells were washed three times with 2 mL Ham’s F-12 medium supplemented with 1 mg/mL BSA and incubated in this medium for 3 h at 37°C to serum starve. The following different ligands were added to specific wells for 5 min: a control of 100 ng/mL EGF purified as described by Qiu et al. (69), the same supernatant samples used for EGF ELISA with an estimated concentration of 100 ng/μL, and a dilution of 1:100 in Ham’s medium for 5 min at 37°C. A G3P2 supernatant sample was used as a nonspecific small protein for a negative control. Wells were washed with ice-cold phosphate-buffered saline and then lysed for 30 min at 4°C in 250 μL of radioimmunoprecipitation assay (RIPA) buffer supplemented with 1 mM activated sodium orthovanadate, a Pierce protease inhibitor minitablet (catalog number A32955; Thermo Fisher Scientific), and Benzonase nuclease (catalog number E1014; Sigma-Aldrich). The total protein concentrations of clarified lysates were determined using the bicinchoninic acid (BCA) assay, and lysates were normalized to the lowest total protein content using RIPA buffer. Normalized amounts of protein lysates were mixed with sample buffer, boiled, separated by 4 to 12% SDS-PAGE, and transferred onto a nitrocellulose membrane. The membrane was blocked with 3% (wt/vol) low-fat milk in TBS, and proteins were detected by incubation with rabbit anti-EGF receptor antibody (D38B1) (catalog number 4267; Cell Signaling), rabbit anti-phospho-EGFR pTyr1068 antibody (catalog number 44-788G; Thermo Fisher Scientific), rabbit anti-β-actin antibody (catalog number 4968; Cell Signaling Technology), and the secondary antibody goat anti-rabbit-680RD antibody (catalog number 926-68071; Li-Cor). Visualization was done as described above in “Western blotting.”
