# Supplementary material for: Export of Diverse and Bioactive Small Proteins through a Type I Secretion System
Source: Appl Environ Microbiol. 2023 Apr 20;89(5):e00335-23. doi: 10.1128/aem.00335-23 (PMC10231218; doi:10.1128/aem.00335-23)
Supplement: Supplemental file 1 — Supplemental material. Download aem.00335-23-s0001.pdf, PDF file, 1.6 MB [file aem.00335-23-s0001.pdf]

Supporting Information for

Export of diverse and bioactive small proteins through a type I secretion system.

Sun-Young Kim[1], Jennifer K. Parker[1], Monica Gonzalez-Magaldi[1], Mady S. Telford[1], Daniel J. Leahy[1], Bryan W. Davies[1,2].

[1]Department of Molecular Biosciences, The University of Texas at Austin, Austin, Texas
[2]John Ring LaMontagne Center for Infectious Diseases, The University of Texas at Austin, Austin, Texas

*Corresponding author: Bryan W. Davies.

Email: bwdavies@utexas.edu

Author Contributions: S-Y.K., J.K.P., M.G-M., M.S.T., D.J.L., and B.W.D. designed research; S-Y.K., M.G-M., and M.S.T. performed research; S-Y.K., J.K.P., M.G-M., M.S.T., D.J.L., and B.W.D. analyzed data; and S-Y.K., J.K.P., M.G-M., D.J.L., and B.W.D. wrote the paper.

Competing Interest Statement: No, the authors declare no competing interest.

Keywords: recombinant-protein production, protein export, type I secretion system, Gram-negative bacteria

This PDF file includes:

Supporting text
References
Figures S1 to S5
Tables S1 to S5

Supporting text

Supplementary Materials and Methods

Calculating small protein properties

We used the ‘‘Peptides’’ R package (1) to calculate charge and hydrophobicity of the random synthetic small proteins, as described in the Peptides package documentation. Using the same package, the amino acid frequencies in a particular class were determined per group and normalized by the total number of amino acids in the group (# of certain amino acids/# of total amino acids) to obtain percent composition. Codon adaptation index of random synthetic small proteins is calculated using CAIcal server (2) and *E. coli* K12 codon usage table (http://www.kazusa.or.jp/codon/).

Pulse-chase assay

G3P2 and G2P3 negative secretion (NS) strains were grown overnight. The cultures were washed with M9 minimal media contains 0.4% (v/v) of glycerol twice and diluted at final OD_{600} = 0.5 in 20 mL of glycerol M9 media with 0.2% (w/v) of arabinose. To pulse the cultures, L-azidohomoalanine (AHA) (Click Chemistry Tools, Cat# 1066-25) was added at 50 μM and incubated for 30 min with shaking. Next, cultures were washed with the glycerol M9 minimal media twice and suspended in 20 mL of glycerol M9 media containing 0.2% (v/v) glucose and 2 mM L-methionine. Before incubation, a pellet from 1 mL of OD_{600} = 0.5 culture was collected (0 min), and pellets from same number of cells (volumes × OD_{600} value) were collected after 5, 10, 15, and 20 min.

Collected pellets were suspended in the same volume of lysis buffer (10 mM Tris-HCl, 100 mM NaCl, 1 mM EDTA, 0.5 mM EGTA, 0.1% (w/v) DOC, 0.5% (w/v) N-lauroylsarcosine, 0.2% (w/v) lysozyme, pH 7.5) supplemented with a Pierce protease inhibitor minitablet (Thermo Fisher Scientific, Cat# A32955) and incubated at 37 °C for 30 min. Samples were sonicated for 10 sec and supernatants were collected by centrifuging at 12,000×g for 10 min. AZDye™ 800 DBCO (Click Chemistry Tools, Cat# 1564-1) was added into 400 μL of lysates at 10 μM and incubated at room temperature for an hour in dark. For immunoprecipitation, Pierce™ Protein A/G Magnetic Beads were coated with anti-V5 antibody (Sigma-Aldrich Cat# V8012), and 25 μL of the beads were added into each incubated lysate sample and incubated at 4 °C with rotation overnight. The beads were washed five times using wash buffer (50 mM HEPES, 500 mM LiCl, 1 mM EDTA, 1% (v/v) NP40, 0.7% (w/v) DOC, pH 7.5), then washed once in phosphate-buffered saline (PBS). To elute precipitated samples, the beads were boiled for 10 min in LDS sample buffer supplemented with 0.05% (v/v) of 2-mercaptoethanol. 4 μL of eluted samples were used

for SDS-PAGE in NuPAGE™ 4–12%, Bis-Tris gel with MES running buffer. Then, the gel was fixed for an hour and visualized by Li-Cor Odyssey Clx Near IR imaging system. Band intensities were measured using Image Studio software (https://www.licor.com/bio/image-studio-lite/).

Recombinant small protein purification

We constructed two pBAD18 derivative plasmids that express recombinant EGF (epidermal growth factor) and Eglin C, respectively. Each recombinant small protein was conjugated with the MccV signal peptide (CvaC15, MRTLTLNELDSVSGG) at the N-terminus, and Strep-tag® II with two glycine residues (GGWSHPQFEK) at the C-terminus. The plasmids were transformed into *E. coli* BL21(DE3) containing pSK01, which constitutively expresses CvaAB, to generate EGF_strep and Eglin C_strep positive secretion strains. Overnight EGF_strep and Eglin C_strep positive secretion cultures were diluted into 0.5 L of fresh M9 minimal media at final OD_{600} = 0.5, with 0.2% (v/v) glycerol and 0.1% (w/v) casamino acids. The cultures were induced with 0.2% (w/v) of arabinose overnight at 30 °C. Supernatants were collected by centrifuging the cultures at 6,000×*g* for 20 min, then filtered through PES 0.22 µm filter membranes (Genesee Scientific, Cat# 25-233). 250 µL bed volumes of Strep-Tactin® XT Sepharose™ (Cytiva, Cat# 29401324) was equilibrated with PBS. Each recombinant small protein in PBS-equilibrated supernatant was bound to the resin and washed once with PBS buffer. Each small protein was eluted using 2 mL of elution buffer (PBS containing 50 mM biotin). Each small protein solution was concentrated by a 3 kDa cut-off ultra-filtration (MilliporeSigma Cat# UFC500324). Purified small proteins were resolved by SDS-PAGE and stained with SimplyBlue™ SafeStain (Thermo Fisher Scientific, Cat# LC6065).

Mass spectrometry

Purified small protein solutions prepared above were desalted using C18 HyperSep™ SpinTip Microscale SPE Extraction Tips (Thermo Fisher Scientific, Cat# 60109-412) and eluted into buffer (60% (v/v) acetonitrile, 1% (v/v) formic acid, and 0.05% (v/v) trifluoroacetic acid). The small protein solutions were directly analyzed by mass spectrometry without further treatments. The mass spectrum of each sample was determined by the UT Austin Center for Biomedical Research Support Biological Mass Spectrometry Facility (RRID:SCR_021728) using a Thermo Orbitrap Fusion Tribrid mass spectrometer. Theoretical masses of small proteins were calculated from https://www.peptidesynthetics.co.uk/tools/.

Small protein quantitative assay

The concentrated small protein solutions prepared as described in “Recombinant small protein purification and SDS-PAGE” were quantified using Pierce™ Quantitative Peptide Assays & Standards (Thermo Fisher Scientific, Cat# 23290) as per manufacture’s protocol.

References

1. Osorio D, Rondón-Villarreal P, Torres Sáez R. 2015. Peptides: A Package for Data Mining of Antimicrobial Peptides. The R Journal 7:4–14.

2. Puigbò P, Bravo IG, Garcia-Vallve S. 2008. CAIcal: A combined set of tools to assess codon usage adaptation. Biol Direct 3:38.

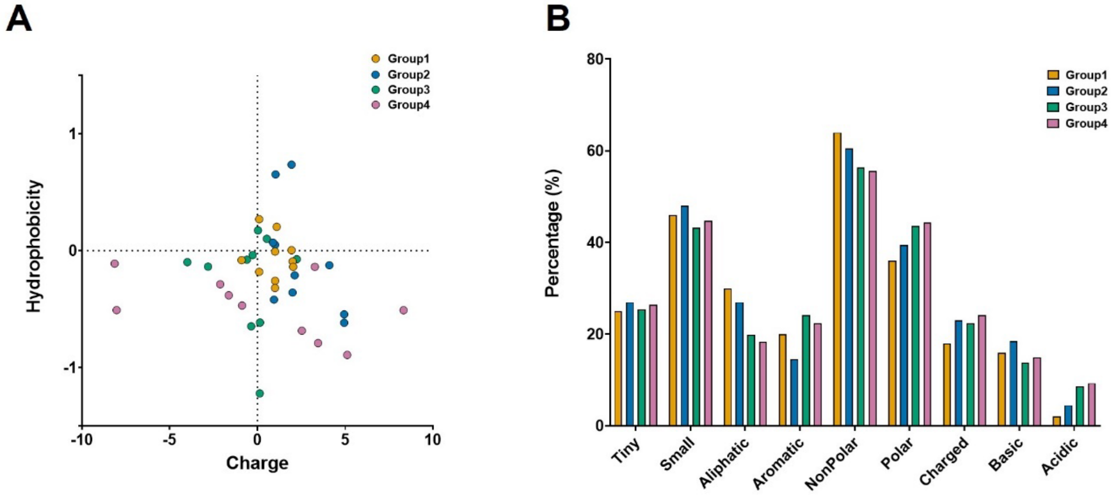

Figure S1. Properties of random synthetic small proteins. (A) Theoretical charge (at pH = 7.0) versus hydrophobicity of small proteins is shown as a scatter plot. Grand average of hydropathicity index (GRAVY) is used to represent the hydrophobicity value of a small protein. Different groups are represented as a different color; Group 1 as orange, Group 2 as blue, Group 3 as green, and Group 4 as purple. (B) The composition of amino acids belonging to a particular class was calculated per group. Amino acids were classified as “Tiny” (alanine, cysteine, glycine, serine, threonine), “Small” (alanine, cysteine, aspartic acid, glycine, asparagine, proline, serine, threonine, valine), “Aliphatic” (alanine, isoleucine, leucine, valine), “Aromatic” (phenylalanine, histidine, tryptophan, tyrosine), “Non-Polar” (alanine, cysteine phenylalanine, glycine, isoleucine leucine, methionine, proline, valine, tryptophan, tyrosine), “Polar” (aspartic acid, glutamic acid, histidine, lysine, asparagine, glutamine, arginine, serine, threonine), “Charged” (aspartic acid, glutamic acid, histidine, lysine, arginine), “Basic” (histidine, lysine, arginine), and “Acidic” (aspartic acid, glutamic acid). Group colors are the same as in (A).

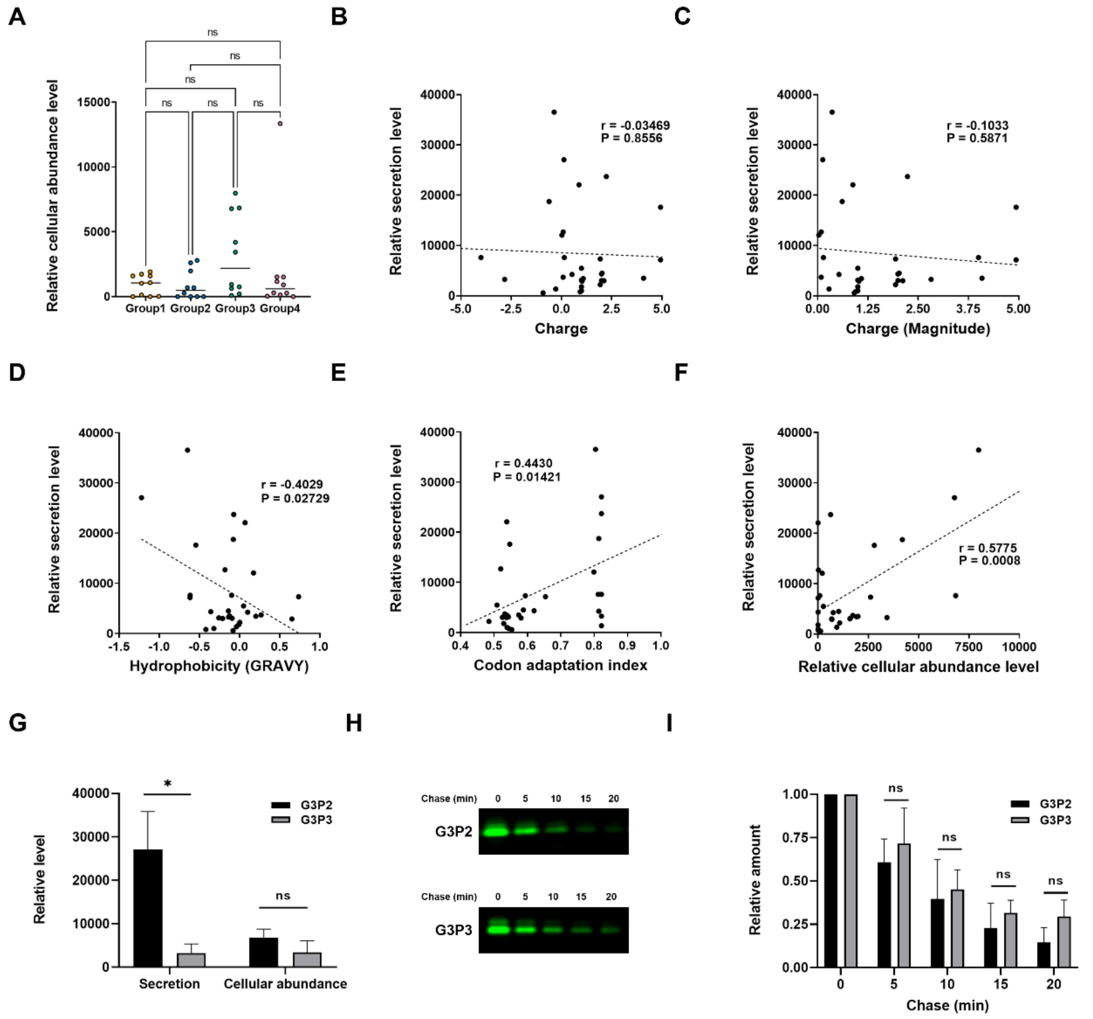

Figure S2. Cellular abundance distribution and analysis of correlation between small protein features and secretion level. (A) The distribution of the cellular abundance level (mean of triplicate) of each group is shown. The median of 10 secretion levels in each group is shown as a bar. Adjusted *P*-values were calculated by ANOVA with Tukey HSD test, and the result is shown (ns = *P* > 0.05). Correlation analysis between the mean of relative secretion level and, (B) charge, (C) magnitude of charge, (D) hydrophobicity (E) codon adaptation index, and (F) the mean of relative cellular abundance was performed. Grand average of hydropathicity index (GRAVY) is used to represent the hydrophobicity value of a small protein. Correlation coefficient (r value) and *P*-value of each analysis is shown. (E) Relative secretion levels and cellular abundance levels of library small protein G3P2 and G3P3 are shown. Two-tailed *P*-values from an unpaired t-test are shown as not significant (ns = *P* > 0.05), or by an asterisk (* = *P* < 0.05). (F) The results of pulse-chase and immunoprecipitation experiments are shown. Chase timepoints are in minutes and the image is a representative of biological triplicate. (G) Relative amount of each small protein at an indicated chase time point is shown. For each small protein in

each replicate, band intensity values were normalized by the intensity value of chase time = 0 min. The mean of biological triplicate is shown with standard deviation. The two small proteins relative amounts are compared by unpaired t-test. Two-tailed *P*-values are shown as not significant (ns = $P > 0.05$).

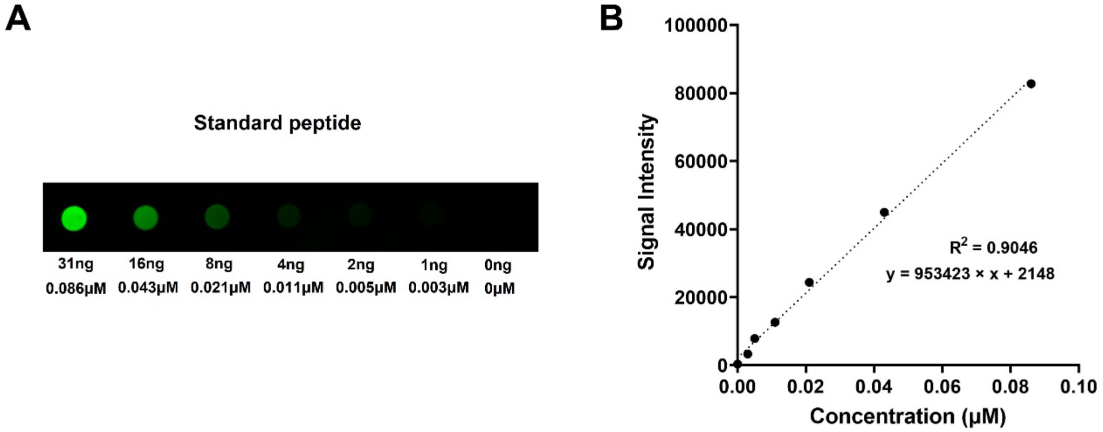

Figure S3. Standard curve of V5-tagged synthetic small protein. (A) Result of dot blot for V5-tagged standard small protein is shown. Standard protein was loaded with serial 2-fold dilution, and the total amount (ng) and concentration (μM) of the protein in each well are shown. A representative dot blot image prepared from a single membrane is shown. (B) The mean of four signal intensity values vs. concentration (μM) of standard protein is plotted. Simple linear regression assay was performed. The best-fit slope is shown as a line with R-squared value and standard curve equation.

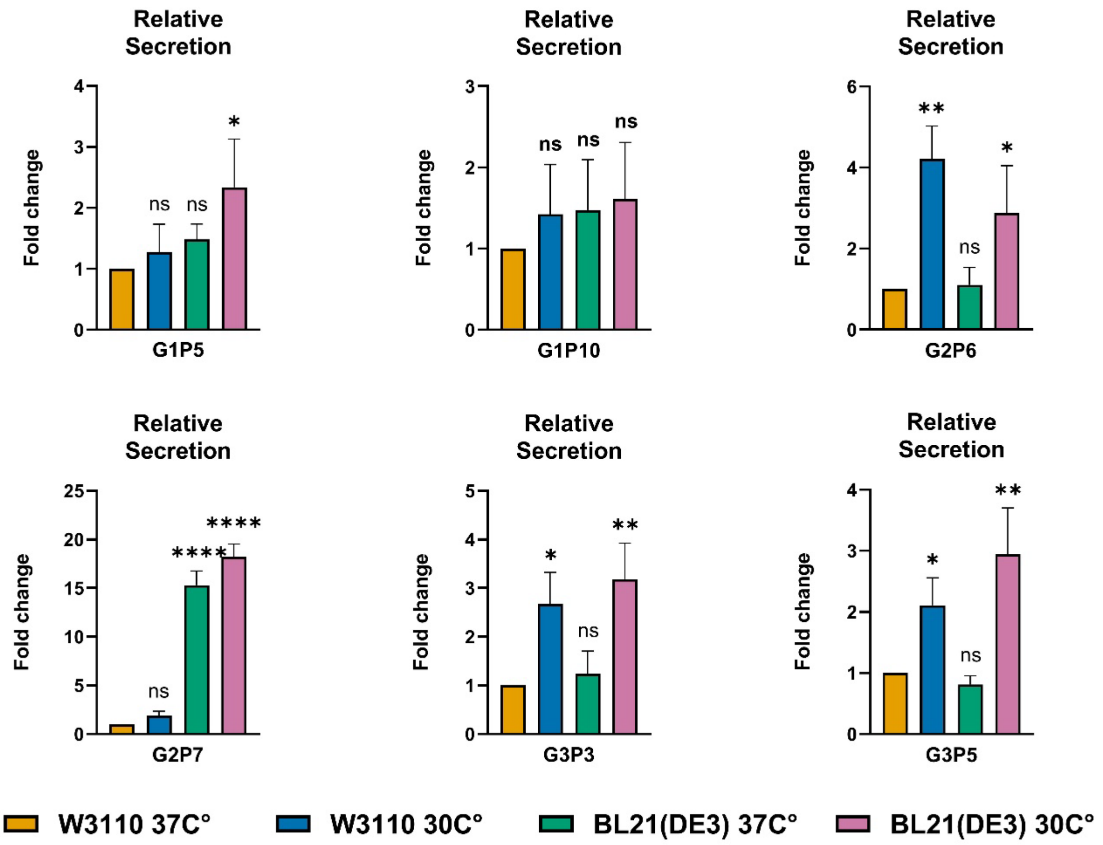

Figure S4. Increased secretion level of random small proteins. Relative secretion levels and cellular abundance levels of random small proteins (G1P5, G1P10, G2P6, G2P7, G3P3 and G3P5) produced from *E. coli* W3110 or BL21(DE3) strains grown at 37 °C or 30 °C (represented as W3110 37 °C: orange, W3110 30 °C: blue, BL21(DE3) 37 °C: green, and BL21(DE3) 30 °C: purple). Small protein levels were normalized to our standard condition (W3110 37 °C), and the results are shown as fold change. Relative secretion levels and cellular abundance levels were calculated as described in “Material and Methods: Dot blot”. The mean of biological triplicate is shown with standard deviation. Adjusted *P*-values were calculated by ANOVA with Dunnett’s multiple comparison test (vs. W3110 37 °C) and are shown as not significant (ns = $P > 0.05$) or the number of asterisks to indicate significance level (* = $P < 0.05$, ** = $P < 0.01$, **** = $P <$ 0.0001).

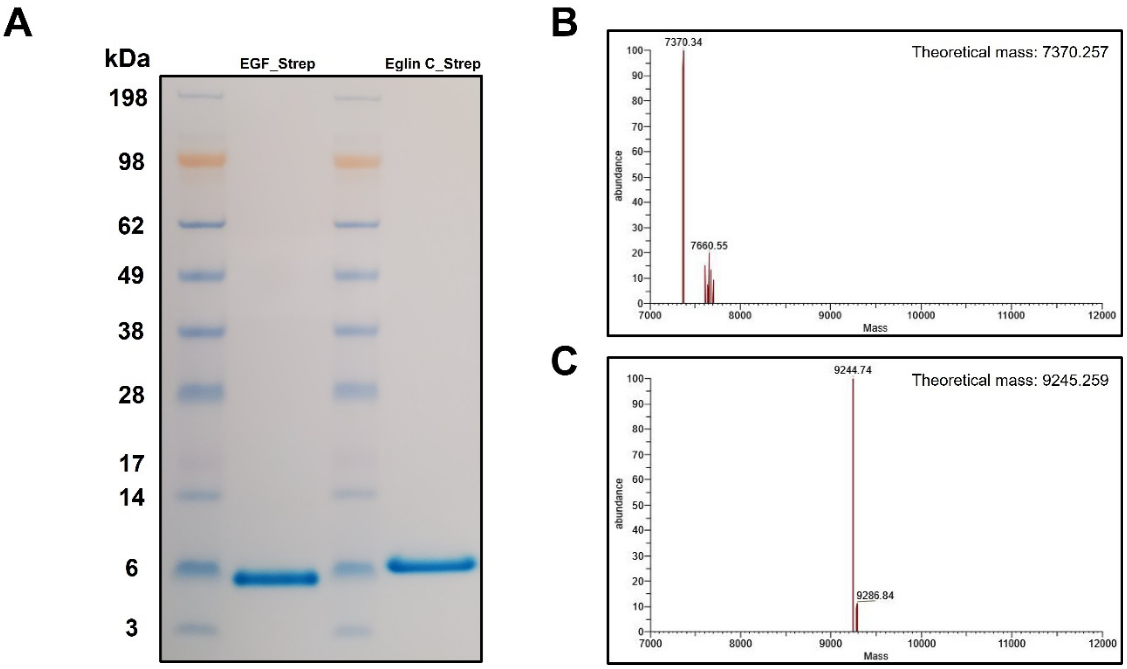

Figure S5. Purification of recombinant small protein from supernatant. (A) The result of SDS-PAGE of strep-tagged EGF (EGF_strep) and Eglin C (Eglin_C strep) is shown. Molecular weights (kDa) of protein ladders are presented on the left. (B and C) Deconvoluted mass spectra of (B) EGF_strep, or (C) Eglin C_strep solution is shown. Average theoretical masses of (B) EGF_strep and (C) Eglin C_strep without the MccV signal peptide are also shown. Cysteine oxidation is considered for the EGF_strep theoretical mass calculation. The y-axis represents the relative abundance (%) calculated from the relative intensity of the detected mass, and the x-axis represents the mass (Da). Averages of deconvoluted masses are shown above the respective peaks.

Table S1. Properties of random synthetic small proteins.

ID	Group	Sequence	Length (aa)	MW	GRAVY	Charge	CAI
G1P1	1	GIGWMLSRARGGGKPIPNPLLGLDST	26	2664.15	-0.09	2	0.53
G1P2	1	IVHRYPRICYGGGKPIPNPLLGLDST	26	2837.37	-0.14	2.03	0.59
G1P3	1	SHSMVSVLVRGGGKPIPNPLLGLDST	26	2632.1	0.2	1.09	0.54
G1P4	1	GIYGHIVVYWGGGKPIPNPLLGLDST	26	2724.18	0.27	0.09	0.53
G1P5	1	SGLRPWMASVGGGKPIPNPLLGLDST	26	2621.08	-0.01	1	0.53
G1P6	1	LSMSICMRPKGGGKPIPNPLLGLDST	26	2683.28	0	1.94	0.49
G1P7	1	VNDRLKLQWVGGGKPIPNPLLGLDST	26	2788.27	-0.26	1	0.54
G1P8	1	PGLIDVSYWHGGGKPIPNPLLGLDST	26	2704.1	-0.08	-0.91	0.55
G1P9	1	SHSVAPWSLQGGGKPIPNPLLGLDST	26	2628.99	-0.18	0.09	0.52
G1P10	1	RYFTLNNFGWGGGKPIPNPLLGLDST	26	2835.24	-0.32	1	0.54
G2P1	2	GSVTRFISFWHMMLLCGMLVGGGKPIPNPLLGLDST	36	3846.7	0.65	1.03	0.58
G2P2	2	SVIRINCLVLVGRLVLGTQVGGGKPIPNPLLGLDST	36	3670.46	0.74	1.94	0.59
G2P3	2	GLWRAFMPWWSFFGVVRDSGGGGKPIPNPLLGLDST	36	3919.56	0.05	1	0.51
G2P4	2	ALLRRDVSFRFWHRSVLYVRGGGKPIPNPLLGLDST	36	4094.82	-0.13	4.09	0.57

G2P5	2	NCDRWRGRWVRFILWFGR GKGGGKPIPNPLLGLDST	36	4126.85	-0.54	4.94	0.55
G2P6	2	HNPRRHWMGLITLKLMSCD LGGGKPIPNPLLGLDST	36	3939.72	-0.21	2.12	0.54
G2P7	2	SVCPAFRVDTFRSTGDKYS NGGGKPIPNPLLGLDST	36	3768.26	-0.42	0.94	0.54
G2P8	2	RQRDRCNPWMPIRAKRSLL VGGGKPIPNPLLGLDST	36	4013.79	-0.62	4.94	0.65
G2P9	2	GNARCSMCEIRPLMVWTVS TGGGKPIPNPLLGLDST	36	3772.48	0.07	0.88	0.54
G2P10	2	GVSYNTMVQRRGGDPARA LMGGGKPIPNPLLGLDST	36	3697.29	-0.36	2	0.62
G3P1	3	LSNGQCNMHCCPCLEYQD YHKHYSNTESFKQLVWMTH ICDNYALSHRAKWGGGKPIP NPLLGLDST	66	7553.66	-0.62	0.14	0.82
G3P2	3	HGVQGINIEKQPKRNNPENE QTRMKMRQERDWSCFAMF HAITRDDSYEQNGGGKPIPN PLLGLDST	66	7583.53	-1.22	0.13	0.82
G3P3	3	ETCHWMELHIPLFETDSFKP YDPKSLDSGHCLYYGFFFK YIGGLHAMCMYGGGKPIPN PLLGLDST	66	7488.72	-0.14	-2.82	0.82
G3P4	3	VQMIWGFCSGWPMITWYA MMFAHIQWAFWNTKISSRG WEFMASAWPEYFVGGGKPI PNPLLGLDST	66	7633	0.17	0.03	0.80
G3P5	3	IMECSTLICTTLDCFICVQGQ WRYCMWNQCWCVCMNWT KTNCAQYSVAKHGGGKPIP NPLLGLDST	66	7466.9	0.1	0.53	0.81
G3P6	3	IICWWPHPQCCWNFEYCFR KNYLTCFQCSEQYSTTFAPV NAFPTIWQIIYGGGKPIPNPL LGLDST	66	7710	-0.04	-0.28	0.82

G3P7	3	GSVIVLDWVQGTNLVHKQH NTINRRHHHSHQEMYPWPT VFEMHGNRIIEEGGGKPIPN PLLGLDST	66	7527.57	-0.65	-0.36	0.80
G3P8	3	HVPKWWYKGFDWTTQVWP YAAMLGFINAHHLDTVMIKIH LGAFHNCDWVEGGGKPIPN PLLGLDST	66	7529.77	-0.08	-0.61	0.81
G3P9	3	VTTFKLWAKALVAFMYDAD HHVNDFLPTLYRVYTTMNIW HFKHKCPCTSYGGGKPIPN PLLGLDST	66	7543.86	-0.07	2.23	0.82
G3P10	3	DDDDELLRLCDNITFFMMCI HEFTMKPWFKTIWFLMCWN AFLNQGSNSTHGGGKPIPN PLLGLDST	66	7625.89	-0.1	-4	0.81
G4P1	4	YMCWHQAINVMEPCAQFY QDIVLSSRVQWQDMDMSM PRLYKMQYVAKSHFSIMYFI HREDIQSSGCCDCNCNPVR KIFCTRYVEMVDCGYWRHF WLPPEEGGGKPIPNPLLGLD ST	116	13715.07	-0.29	-2.13	0.89
G4P2	4	FIIYIPKVYDWYAASMGCTY PDGLGFRMVSMRVIWYAW YVYSCYTAVETNKFGDCGC GNYNHPDQEQIRYKTSEQE STKMFFRWAPEWQNNHRH HLMVEMGGGKPIPNPLLGL DST	116	13608.64	-0.47	-0.88	0.89
G4P3	4	HSYCEWLMAKILSMGEQW WHKYYFGLRHQFNVSKGYH FNTSFDFHRCGEPKNNYYA RTHCEKEYSNDDVHPRQNS MGRWGAEWPALIFKPLFGI NKWGNFGGGKPIPNPLLGL DST	116	13688.57	-0.79	3.45	0.88
G4P4	4	SGIYCQITRWVHPFSESTQN MDNMANTKRKPQWHYPRR HQHKEAQFVFFGIARGWPF	116	13624.55	-0.69	2.52	0.89

		QFFEQGWVTIDVHEEHLWIF<br>CFGERNMEHGNVERAPTIS<br>SRIKGGGKPIPNPLLGLDST					
G4P5	4	KHWKQRHVYYRQAVYQQQ<br>KCQMYNVPYSPASGCFNC<br>QPNCHRKDFDWRDHTGYC<br>FKMLQFHNFITPKRCTESAL<br>FAGQECRPDFAQESGAQKD<br>FSGMHPGGGGKPIPNPLLG<br>LDST	116	13393.27	-0.89	5.11	0.90
G4P6	4	FNPESSHQPSTIPKSHFRIIC<br>HFVRDWHFPCGSWTFSVV<br>DIYFCEMYTTLGNPHGFVIC<br>CTYGSQYSGDNRCADKLER<br>HPAMMENTYGWHGHTSAG<br>LAQPGGGKPIPNPLLGLDST	116	12962.73	-0.38	-1.64	0.90
G4P7	4	DKMEPQWNHSPRCFASLC<br>CGGSHTMMAWNHVISWKG<br>RDLVIGVNRHCATPPHSQF<br>WHNAWWQGFIKHVIETPRL<br>GNMAKNMQCFAACALAVAF<br>PVDISQLGGGKPIPNPLLGL<br>DST	116	12878.07	-0.14	3.27	0.89
G4P8	4	RGKWMMCNTHMAHWNTYF<br>WDGSAVHLTDDFYRNGPAK<br>SYNLFMVQNHKESRHNYKV<br>CFFCYLITTYITIAKHKRMNE<br>NYWWMAQVYLKFVRWHAR<br>NCCYAGGGKPIPNPLLGLDS<br>T	116	13910.21	-0.51	8.32	0.89
G4P9	4	VMQGDTWWECEPSEAEIQ<br>MLYWPWGSQKDPIDWAYL<br>CDTWKYTGDLCSGGPEQP<br>DEHRIHDAIGRAFYRPCPSL<br>NMYYLSQRWAIFDTHNSLA<br>AGSYCFMGGGKPIPNPLLG<br>LDST	116	13255.01	-0.51	-8.03	0.89
G4P10	4	TGRQVTEVTVWHALTTICGI<br>SELEFTYGACPMWENMELE<br>KFSGNVCYELQDHCFCDW<br>WQYTERCLENLPMIELPIQW	116	13468.68	-0.11	-8.15	0.89

		KPFTLHEWWIIGRCPLTIMN SWAGGGKPIPNPLLGLDST					

* aa: amino acids, GRAVY: Grand average of hydropathy, MW: Molecular weight (g/mol), CAI: Codon Adaptation Index.

Table S2. Selected bioactive small proteins.

Name	Function	Origin	Length (aa)	MW
Pediocin PA-1	Antibacterial, strongly inhibits *L. monocytogenes*	*Pediococcus acidilactici*	44	4628.19
α-factor	Pheromone, arrests cell cycle	*Saccharomyces cerevisiae*	13	1683.99
Eglin C	Protease inhibitor, inhibits neutrophil elastase	*Hirudo medicinalis*	70	8091.05
EGF	Epidermal growth factor, activates EGFR signaling	*Homo sapiens*	53	6353.21

* aa: amino acids, MW: Molecular weight (g/mol).

Table S3. Bacterial and yeast strains.

Name	Description	Source
Escherichia coli DH5α	*fhuA2 lac(del)U169 phoA glnV44 Φ80' lacZ(del)M15 gyrA96 recA1 relA1 endA1 thi-1 hsdR17*	NEB®
Escherichia coli W3110	Wild-type	Lab stock
Escherichia coli BL21(DE3)	*fhuA2 [lon] ompT gal (λ DE3) [dcm] ∆hsdS* *λ DE3 = λ sBamHIo ∆EcoRI-B int::(lacI::PlacUV5::T7 gene1) i21 ∆nin5*	NEB®
Escherichia coli SM10 (λpir)	*thi thr leu tonA lacY supE recA*::RP4-2-Tc::Mu Km *λpir*	Lab stock
Escherichia coli Nissle 1917	Wild-type, Probiotic strain	Lab stock
Salmonella enterica Ty21a	CDC 2861-79	ATCC® 33459
Vibrio cholerae CVD103-HgR	mutant from clinical isolate 569B	ATCC® 55456
Listeria monocytogenes EGD-e	Wild-type	Lab stock
Sacharomyces cerevisea CMY 740-1D	*MATa his3Δ1 leu2-3,112 trp1-289 ura3-52 bar1::loxP*	Matouschek Lab
SK00	*E. coli* W3110, pBAD18-Km/pACYC184	This study
SK01	*E. coli* W3110, MccV positive secretion, encoding pSKP00/pSK01	This study
SK02	*E. coli* W3110, MccV negative secretion, encoding pSKP00/pACYC184	This study
SK03	*E. coli* W3110, MccV protease-deficient secretion, encoding pSKP00/pSK02	This study
SK04	*E. coli* W3110, MccV_V5 positive secretion, encoding pSKP01/pSK01	This study
SK05	*E. coli* W3110, MccV_V5 negative secretion, encoding pSKP01/pACYC184	This study
SK06	*E. coli* W3110, MccV_V5 protease-deficient secretion, encoding pSKP01/pSK02	This study
SK07	*E. coli* W3110, G1P1 positive secretion, encoding pSKP02/pSK01	This study
SK08	*E. coli* W3110, G1P1 negative secretion, encoding pSKP02/pACYC184	This study
SK09	*E. coli* W3110, G1P2 positive secretion, encoding pSKP03/pSK01	This study
SK10	*E. coli* W3110, G1P2 negative secretion, encoding pSKP03/pACYC184	This study
SK11	*E. coli* W3110, G1P3 positive secretion, encoding pSKP04/pSK01	This study
SK12	*E. coli* W3110, G1P3 negative secretion, encoding pSKP04/pACYC184	This study
SK13	*E. coli* W3110, G1P4 positive secretion, encoding pSKP05/pSK01	This study
SK14	*E. coli* W3110, G1P4 negative secretion, encoding pSKP05/pACYC184	This study
SK15	*E. coli* W3110, G1P5 positive secretion, encoding pSKP06/pSK01	This study

SK16	*E. coli* W3110, G1P5 negative secretion, encoding pSKP06/pACYC184	This study
SK17	*E. coli* W3110, G1P6 positive secretion, encoding pSKP07/pSK01	This study
SK18	*E. coli* W3110, G1P6 negative secretion, encoding pSKP07/pACYC184	This study
SK19	*E. coli* W3110, G1P7 positive secretion, encoding pSKP08/pSK01	This study
SK20	*E. coli* W3110, G1P7 negative secretion, encoding pSKP08/pACYC184	This study
SK21	*E. coli* W3110, G1P8 positive secretion, encoding pSKP09/pSK01	This study
SK22	*E. coli* W3110, G1P8 negative secretion, encoding pSKP09/pACYC184	This study
SK23	*E. coli* W3110, G1P9 positive secretion, encoding pSKP10/pSK01	This study
SK24	*E. coli* W3110, G1P9 negative secretion, encoding pSKP10/pACYC184	This study
SK25	*E. coli* W3110, G1P10 positive secretion, encoding pSKP11/pSK01	This study
SK26	*E. coli* W3110, G1P10 negative secretion, encoding pSKP11/pACYC184	This study
SK27	*E. coli* W3110, G2P1 positive secretion, encoding pSKP12/pSK01	This study
SK28	*E. coli* W3110, G2P1 negative secretion, encoding pSKP12/pACYC184	This study
SK29	*E. coli* W3110, G2P2 positive secretion, encoding pSKP13/pSK01	This study
SK30	*E. coli* W3110, G2P2 negative secretion, encoding pSKP13/pACYC184	This study
SK31	*E. coli* W3110, G2P3 positive secretion, encoding pSKP14/pSK01	This study
SK32	*E. coli* W3110, G2P3 negative secretion, encoding pSKP14/pACYC184	This study
SK33	*E. coli* W3110, G2P4 positive secretion, encoding pSKP15/pSK01	This study
SK34	*E. coli* W3110, G2P4 negative secretion, encoding pSKP15/pACYC184	This study
SK35	*E. coli* W3110, G2P5 positive secretion, encoding pSKP16/pSK01	This study
SK36	*E. coli* W3110, G2P5 negative secretion, encoding pSKP16/pACYC184	This study
SK37	*E. coli* W3110, G2P6 positive secretion, encoding pSKP17/pSK01	This study
SK38	*E. coli* W3110, G2P6 negative secretion, encoding pSKP17/pACYC184	This study
SK39	*E. coli* W3110, G2P7 positive secretion, encoding pSKP18/pSK01	This study
SK40	*E. coli* W3110, G2P7 negative secretion, encoding pSKP18/pACYC184	This study
SK41	*E. coli* W3110, G2P8 positive secretion, encoding pSKP19/pSK01	This study
SK42	*E. coli* W3110, G2P8 negative secretion, encoding pSKP19/pACYC184	This study

SK43	*E. coli* W3110, G2P9 positive secretion, encoding pSKP20/pSK01	This study
SK44	*E. coli* W3110, G2P9 negative secretion, encoding pSKP20/pACYC184	This study
SK45	*E. coli* W3110, G2P10 positive secretion, encoding pSKP21/pSK01	This study
SK46	*E. coli* W3110, G2P10 negative secretion, encoding pSKP21/pACYC184	This study
SK47	*E. coli* W3110, G3P1 positive secretion, encoding pSKP22/pSK01	This study
SK48	*E. coli* W3110, G3P1 negative secretion, encoding pSKP22/pACYC184	This study
SK49	*E. coli* W3110, G3P2 positive secretion, encoding pSKP23/pSK01	This study
SK50	*E. coli* W3110, G3P2 negative secretion, encoding pSKP23/pACYC184	This study
SK51	*E. coli* W3110, G3P3 positive secretion, encoding pSKP24/pSK01	This study
SK52	*E. coli* W3110, G3P3 negative secretion, encoding pSKP24/pACYC184	This study
SK53	*E. coli* W3110, G3P4 positive secretion, encoding pSKP25/pSK01	This study
SK54	*E. coli* W3110, G3P4 negative secretion, encoding pSKP25/pACYC184	This study
SK55	*E. coli* W3110, G3P5 positive secretion, encoding pSKP26/pSK01	This study
SK56	*E. coli* W3110, G3P5 negative secretion, encoding pSKP26/pACYC184	This study
SK57	*E. coli* W3110, G3P6 positive secretion, encoding pSKP27/pSK01	This study
SK58	*E. coli* W3110, G3P6 negative secretion, encoding pSKP27/pACYC184	This study
SK59	*E. coli* W3110, G3P7 positive secretion, encoding pSKP28/pSK01	This study
SK60	*E. coli* W3110, G3P7 negative secretion, encoding pSKP28/pACYC184	This study
SK61	*E. coli* W3110, G3P8 positive secretion, encoding pSKP29/pSK01	This study
SK62	*E. coli* W3110, G3P8 negative secretion, encoding pSKP29/pACYC184	This study
SK63	*E. coli* W3110, G3P9 positive secretion, encoding pSKP30/pSK01	This study
SK64	*E. coli* W3110, G3P9 negative secretion, encoding pSKP30/pACYC184	This study
SK65	*E. coli* W3110, G3P10 positive secretion, encoding pSKP31/pSK01	This study
SK66	*E. coli* W3110, G3P10 negative secretion, encoding pSKP31/pACYC184	This study
SK67	*E. coli* W3110, G4P1 positive secretion, encoding pSKP32/pSK01	This study
SK68	*E. coli* W3110, G4P1 negative secretion, encoding pSKP32/pACYC184	This study
SK69	*E. coli* W3110, G4P2 positive secretion, encoding pSKP33/pSK01	This study

SK70	*E. coli* W3110, G4P2 negative secretion, encoding pSKP33/pACYC184	This study
SK71	*E. coli* W3110, G4P3 positive secretion, encoding pSKP34/pSK01	This study
SK72	*E. coli* W3110, G4P3 negative secretion, encoding pSKP34/pACYC184	This study
SK73	*E. coli* W3110, G4P4 positive secretion, encoding pSKP35/pSK01	This study
SK74	*E. coli* W3110, G4P4 negative secretion, encoding pSKP35/pACYC184	This study
SK75	*E. coli* W3110, G4P5 positive secretion, encoding pSKP36/pSK01	This study
SK76	*E. coli* W3110, G4P5 negative secretion, encoding pSKP36/pACYC184	This study
SK77	*E. coli* W3110, G4P6 positive secretion, encoding pSKP37/pSK01	This study
SK78	*E. coli* W3110, G4P6 negative secretion, encoding pSKP37/pACYC184	This study
SK79	*E. coli* W3110, G4P7 positive secretion, encoding pSKP38/pSK01	This study
SK80	*E. coli* W3110, G4P7 negative secretion, encoding pSKP38/pACYC184	This study
SK81	*E. coli* W3110, G4P8 positive secretion, encoding pSKP39/pSK01	This study
SK82	*E. coli* W3110, G4P8 negative secretion, encoding pSKP39/pACYC184	This study
SK83	*E. coli* W3110, G4P9 positive secretion, encoding pSKP40/pSK01	This study
SK84	*E. coli* W3110, G4P9 negative secretion, encoding pSKP40/pACYC184	This study
SK85	*E. coli* W3110, G4P10 positive secretion, encoding pSKP41/pSK01	This study
SK86	*E. coli* W3110, G4P10 negative secretion, encoding pSKP41/pACYC184	This study
SK87	*E. coli* DH5α, codon optimized G1P6 positive secretion, encoding pSKP42/pSK01	This study
SK88	*E. coli* DH5α, codon optimized G1P6 negative secretion, encoding pSKP42/pACYC184	This study
SK89	*E. coli* DH5α, G1P6_2X positive secretion, encoding pSKP43/pSK01	This study
SK90	*E. coli* DH5α, G1P6 _2X negative secretion, encoding pSKP43/pACYC184	This study
SK91	*E. coli* DH5α, G3P2 positive secretion, encoding pSKP23/pSK01	This study
SK92	*E. coli* DH5α, G3P2 negative secretion, encoding pSKP23/pACYC184	This study
SK93	*E. coli* DH5α, G3P2_2X positive secretion, encoding pSKP44/pSK01	This study
SK94	*E. coli* DH5α, G3P2_2X negative secretion, encoding pSKP44/pACYC184	This study
SK95	*E. coli* W3110, Pediocin PA-1 positive secretion, encoding pSKP45/pSK01	This study

SK96	*E. coli* W3110, Pediocin PA-1 negative secretion, encoding pSKP45/pACYC184	This study
SK97	*E. coli* W3110, Pediocin PA-1 protease-deficient secretion, encoding pSKP45/pSK02	This study
SK98	*E. coli* W3110, α-factor positive secretion, encoding pSKP46/pSK01	This study
SK99	*E. coli* W3110, α-factor negative secretion, encoding pSKP46/pACYC184	This study
SK100	*E. coli* W3110, α-factor protease-deficient secretion, encoding pSKP46/pSK02	This study
SK101	*E. coli* W3110, eglin C positive secretion, encoding pSKP47/pSK01	This study
SK102	*E. coli* W3110, eglin C protease-deficient secretion, encoding pSKP47/pSK02	This study
SK103	*E. coli* W3110, EGF positive secretion, encoding pSKP48/pSK01	This study
SK104	*E. coli* W3110, EGF negative secretion, encoding pSKP48/pACYC184	This study
SK105	*E. coli* Nissle 1917, Pediocin PA-1 positive secretion, encoding pSKP49	This study
SK106	*E. coli* Nissle 1917, Pediocin PA-1 negative secretion, encoding pSKP50	This study
SK107	*Salmonella enterica* Ty21a, Pediocin PA-1 positive secretion, encoding pSKP49	This study
SK108	*Salmonella enterica* Ty21a, Pediocin PA-1 negative secretion, encoding pSKP50	This study
SK109	*Vibrio cholerae* CVD103-HgR, Pediocin PA-1 positive secretion, encoding pSKP49	This study
SK110	*Vibrio cholerae* CVD103-HgR, Pediocin PA-1 negative secretion, encoding pSKP50	This study
SK111	*E. coli* BL21(DE3), empty vector, encoding pBAD18-Km/pACYC184	This study
SK112	*E. coli* BL21(DE3), G1P5 positive secretion, encoding pSKP06/pSK01	This study
SK113	*E. coli* BL21(DE3), G1P10 positive secretion, encoding pSKP11/pSK01	This study
SK114	*E. coli* BL21(DE3), G2P6 positive secretion, encoding pSKP17/pSK01	This study
SK115	*E. coli* BL21(DE3), G2P7 positive secretion, encoding pSKP18/pSK01	This study
SK116	*E. coli* BL21(DE3), G3P3 positive secretion, encoding pSKP24/pSK01	This study
SK117	*E. coli* BL21(DE3), G3P5 positive secretion, encoding pSKP26/pSK01	This study
SK118	*E. coli* BL21(DE3), G1P5 negative secretion, encoding pSKP06/pACYC184	This study
SK119	*E. coli* BL21(DE3), G1P10 negative secretion, encoding pSKP11/pACYC184	This study
SK120	*E. coli* BL21(DE3), G2P6 negative secretion, encoding pSKP17/pACYC184	This study
SK121	*E. coli* BL21(DE3), G2P7 negative secretion, encoding pSKP18/pACYC184	This study

SK122	*E. coli* BL21(DE3), G3P3 negative secretion, encoding pSKP24/pACYC184	This study
SK123	*E. coli* BL21(DE3), G3P5 negative secretion, encoding pSKP26/pACYC184	This study
SK124	*E. coli* BL21(DE3), EGF_strep positive secretion, encoding pSKP51/pSK01	This study
SK125	*E. coli* BL21(DE3), Eglin C_strep positive secretion, encoding pSKP52/pSK01	This study

Table S4. Primers and gBlocks.

Type	Name	Sequence	Usage
primer	random_20mer_V5	ATAGAGCTCGAATTCAGGAGGAAACGATGAGAACTCTGACTCTAAATGAATTAGATTCTGTTTCTGGTGGTNNKNNKNNKNNKNNKNNKNNKNNKNNKNNKNNKNNKNNKNNKNNKNNKNNKNNKNNKNNKGGAGGAGGTAAACCTATTCCTAATCCTCTCCTAGGTTTAGATTCTACTTAAGTCGACAGGAGGAAACGA	template for generating group2 small protein ORFs
primer	cvaC15_induce_F	ATAGAGCTCGAATTCAGGAGGAAACGATGAGAACTCTGACTCTAAATG	amplifying DNA containing MccV signal peptide sequences
primer	V5_R	TATGTCGACTTAAGTAGAATCTAAACCTAGGAGAGG	amplifying DNA containing V5-tag sequences
primer	cvaB_mutation_F	CATCAGACGGAGACCGCTGAATCTGGACTG	to make pSK02
primer	cvaB_mutation_R	GAGCCCGGGTGGCCATCC	to make pSK02
primer	pBAD_vecF	GTCTGCTTACATAAACAGTAATACAAGGGGTG	to delete Sfo I site of pBAD_cvi_cvaC
primer	pBAD_vecR	GCGCCACAGGTGCGGTTGCTATCTCCTTGCTGCCTCGCG	to delete Sfo I site of pBAD_cvi_cvaC
primer	pBAD_fragF	GCGCGAGGCAGCAAGGAGATAGCAACCGCACCTGTGG	to delete Sfo I site of pBAD_cvi_cvaC
primer	pBAD_fragR	GCTCATAACACCCCTTGTATTACTGTTTATGTAAGCAGACAGTTTTATTGTTCATGA	to delete Sfo I site of pBAD_cvi_cvaC
gBlock	pBAD_MCS_2	ATAGAGCTCGAATTCAGGAGGAAACGATGAGAACTCTGACTCTAAATGAATTAGATTCTGTTTCTGGCGCCGGTACCCGGGGATCCTCTAGAGTCGACCTGCAGGCATGCAAGCTTGGCTGTTTTGGCGGATGAGAGAAGATTTTCAGCCTGATACAGATTAAATCAGAACGCAGAAGCGGTCTGATAAAACAGAATTTGCCTGGCGGCAGTAGCGCGGTGGTCCCACCTGACCCCATGCCGAACTCAGAAGTGAAACGCCGTAGCGCCGATGGTAGTGTGGACTAGTGGTCTCCCCATGCATA	template for generating pSK00 (MccV signal peptide, sfoI site containing)
primer	pBAD_MCS_2_R	TATGCATGGGGAGACCACTAGTCCACACT	to generate pSK00 or amplify the region
gBlock	EglinC	GAGCTCGAATTCAGGAGGAAACGATGAGAACTCTGACTCTAAATGAATTAGATTCTGTTTCTGGTGGTACCGAATTTGGCAGCGAACTGAAAAGCTTTCCGGAAGTGGTGGGCAAAACCGTGGATCAGGCGCGCGAATATTTTACCCTGCATTATCCGCAGTATGATGTGTATTTTCTGCCGGAAGGCAGCCCGGTGACCCTGGATCTGCGCTATAACCGCGTGCGCGTGTTTTATAACCCGGG	to clone eglinC into pBAD

		CACCAACGTGGTGAACCATGTGCCGCATGTGGGCGGAGGAGGTAAACCTATTCCTAATCCTCTCCTAGGTTTAGATTCTACTTAAAAGTCGAC	
primer	EglinC_R	ATAGTCGACTTAGCCCACATGCGGCACATGGT	to amplify eglinC
primer	pBAD_N10mer	ATCCCGGGNNKNNKNNKNNKNNKNNKNNKNNKNNKNNKGGAGGAGGTAAACCTATTCCTAATCCTCTCCTAGGT	to generating group1 small protein ORFs
primer	G3P2_bbs1	ATAGAGCTCGAAGACCATGGCGTGCAGGGC	to generate BbsI site containing G3P2
primer	G3P2_bbs2	ATGAAGACAAGCCATGGTTCTGTTCATAGCTATCATCGCGG	to generate G3P2_2X via SacI and BbsI cloning
gBlock	Pediocin PA-1	AAATACTACGGCAATGGGGTGACCTGTGGGAAACATTCCTGCTCCGTTGACTGGGGGAAAGCGACCACCTGTATCATCAATAACGGAGCGATGGCCTGGGCTACGGGCGGTCACCAGGGCAATCACAAGTGT	template for Pediocin PA-1
primer	Pediocin_F	ATAGAGCTCAGGAGGAAACGATGAGAACTCTGACTCTAAATGAATTAGATTCTGTTTCTGGTGGTAAATACTACGGCAATGGTGTAACG	amplifying Pediocin PA-1
primer	Pediocin_F	ATATCTAGATTAGCACTTATGATTTCCCTGGTGGC	amplifying Pediocin PA-1
primer	alpha_F	ATCCCGGGTGGCACTGGCTGCAGCTGAAACCGGGTCAGCCGATGTACTAAGGTACCTGGGGATCCTCTAGAG	amplifying alpha-factor
gBlock	EGF	ATACCCGGGAACAGCGATAGCGAATGCCCGCTGAGCCATGATGGCTATTGCCTGCATGATGGCGTGTGCATGTATATTGAAGCGCTGGATAAATATGCGTGCAACTGCGTGGTGGGCTATATTGGCGAACGCTGCCAGTATCGCGATCTGAAATGGTGGGAACTGCGCTAAGTCGACATA	template for EGF
primer	EGF_F	ATACCCGGGAACAGCGATAG	to generate EGF
primer	EGF_R	TATGTCGACTTAGCGCAGTTCCC	to generate EGF
gBlock	Random1	ACACTTTGCTATGCCCGGGTATATGTGCTGGCATCAGGCGATTAACGTGATGGAACCGTGCGCGCAGTTTTATCAGGATATTGTGCTGAGCAGCCGCGTGCAGTGGCAGGATATGGATATGAGCATGCCGCGCCTGTATAAAATGCAGTATGTGGCGAAAAGCCATTTTAGCATTATGTATTTTATTCATCGCGAAGATATTCAGAGCAGCGGCTGCTGCGATTGCAACTGCAACCCGGTGCGCAAAATTTTTTGCACCCGCTATGTGGAAATGGTGGATTGCGGCTATTGGCGCCATTTTTGGCTG	template for G3P1 and G4P1

		CCGCCGGAAGAAGGAGGAGGTAAACCTATT CCTAATCCCGGGCTGAGCAACGGCCAGTGC AACATGCATTGCTGCCCGTGCCTGGAATATC AGGATTATCATAAACATTATAGCAACACCGA AAGCTTTAAACAGCTGGTGTGGATGACCCAT ATTTGCGATAACTATGCGCTGAGCCATCGCG CGAAATGGGGAGGACGTGATGTCTTC	
gBlock	Random2	ACACTTTGCTATGCCCGGGTTTATTATTTATA TTCCGAAAGTGTATGATTGGTATGCGGCGAG CATGGGCTGCACCTATCCGGATGGCCTGGG CTTTCGCATGGTGAGCATGCGCGTGATTTG GTATGCGTGGTATGTGTATAGCTGCTATACC GCGGTGGAAACCAACAAATTTGGCGATTGC GGCTGCGGCAACTATAACCATCCGGATCAG GAACAGATTCGCTATAAAACCAGCGAACAGG AAAGCACCAAAATGTTTTTTCGCTGGGCGCC GGAATGGCAGAACAACCACAGGCACCACCT GATGGTGGAAATGGGAGGAGGTAAACCTAT TCCTAATCCCGGGCATGGCGTGCAGGGCAT TAACATTGAAAAACAGCCGAAACGCAACAAC CCGGAAAACGAACAGACCCGCATGAAAATG CGCCAGGAACGCGATTGGAGCTGCTTTGCG ATGTTTCATGCGATTACCCGCGATGATAGCT ATGAACAGAAC	template for G3P2 and G4P2
gBlock	Random3	ACACTTTGCTATGCCCGGGCACAGTTATTGC GAATGGCTGATGGCGAAAATTCTGAGCATG GGCGAACAGTGGTGGCATAAATATTATTTTG GCCTGCGCCATCAGTTTAACGTGAGCAAAG GCTATCATTTTAACACCAGCTTTGATTTTCAT CGCTGCGGCGAACCGAAAAACAACTATTATG CGCGCACCCATTGCGAAAAAGAATATAGCAA CGATGATGTGCATCCGCGCCAGAACAGCAT GGGCCGCTGGGGCGCGGAATGGCCGGCGC TGATTTTTAAACCGCTGTTTGGCATTAACAAA TGGGGCAACTTTGGAGGAGGTAAACCTATTC CTAATCCCGGGGAAACCTGCCATTGGATGG AACTGCATATTCCGCTGTTTGAAACCGATAG CTTTAAACCGTATGATCCGAAAAGCCTGGAT AGCGGCCATTGCCTGTATTATGGCTTTTTTTT TAAATATATTGGCGGCCTGCATGCGATGTGC ATGTATGGAGGACGTGATGTCTTC	template for G3P3 and G4P3
gBlock	Random4	ACACTTTGCTATGCCCGGGAGCGGCATTTAT TGCCAGATTACCCGCTGGGTGCATCCGTTTA GCGAAAGCACCCAGAACATGGATAACATGG CGAACACCAAACGCAAACCGCAGTGGCATT ATCCGCGCCGCCATCAGCATAAAGAAGCGC	template for G3P4 and G4P4

		AGTTTGTGTTTTTTGGCATTGCGCGCGGCTG<br>GCCGTTTCAGTTTTTTGAACAGGGCTGGGTG<br>ACCATTGATGTGCATGAAGAACATCTGTGGA<br>TTTTTTGCTTTGGCGAACGCAACATGGAACA<br>TGGCAACGTGGAACGCGCGCCGACCATTAG<br>CAGCCGCATTAAAGGAGGAGGTAAACCTATT<br>CCTAATCCCGGGGTGCAGATGATTTGGGGC<br>TTTTGCAGCGGCTGGCCGATGATTACCTGGT<br>ATGCGATGATGTTTGCGCATATTCAGTGGGC<br>GTTTTGGAACACCAAAATTAGCAGCCGCGG<br>CTGGGAATTTATGGCGAGCGCGTGGCCGGA<br>ATATTTTGTGGGAGGACGTGATGTCTTC	
gBlock	Random5	ACACTTTGCTATGCCCGGGAAACATTGGAAA<br>CAGCGCCATGTGTATTATCGCCAGGCGGTG<br>TATCAGCAGCAGAAATGCCAGATGTATAACG<br>TGCCGTATAGCCCGGCGAGCGGCTGCTTTA<br>ACTGCCAGCCGAACTGCCATCGCAAAGATTT<br>TGATTGGCGCGATCATACCGGCTATTGCTTT<br>AAAATGCTGCAGTTTCATAACTTTATTACCCC<br>GAAACGCTGCACCGAAAGCGCGCTGTTTGC<br>GGGCCAGGAATGCCGCCCGGATTTTGCGCA<br>GGAAAGCGGCGCGCAGAAAGATTTTAGCGG<br>CATGCATCCGGGCGGAGGAGGTAAACCTAT<br>TCCTAATCCCGGGATTATGGAATGCAGCACC<br>CTGATTTGCACCACCCTGGATTGCTTTATTT<br>GCGTGCAGGGCCAGTGGCGCTATTGCATGT<br>GGAACCAGTGCTGGTGCGTGTGCATGAACT<br>GGACCAAAACCAACTGCGCGCAGTATAGCG<br>TGGCGAAACATGGAGGACGTGATGTCTTC	template for G3P5 and G4P5
gBlock	Random6	ACACTTTGCTATGCCCGGGTTTAACCCGGAA<br>AGCAGCCATCAGCCGAGCACCATTCCGAAA<br>AGCCATTTTCGCATTATTTGCCATTTTGTGCG<br>CGATTGGCATTTTCCGTGCGGCAGCTGGAC<br>CTTTAGCGTGGTGGATATTTATTTTTGCGAAA<br>TGTATACCACCCTGGGCAACCCGCATGGCT<br>TTGTGATTTGCTGCACCTATGGCAGCCAGTA<br>TAGCGGCGATAACCGCTGCGCGGATAAACT<br>GGAACGCCATCCGGCGATGATGGAAAACAC<br>CTATGGCTGGCATGGCCATACCAGCGCGGG<br>CCTGGCGCAGCCGGGAGGAGGTAAACCTAT<br>TCCTAATCCCGGGATTATTTGCTGGTGGCCG<br>CATCCGCAGTGCTGCTGGAACTTTGAATATT<br>GCTTTCGCAAAAACTATCTGACCTGCTTTCA<br>GTGCAGCGAACAGTATAGCACCACCTTTGC<br>GCCGGTGAACGCGTTTCCGACCATTTGGCA<br>GATTATTTATGGAGGACGTGATGTCTTC	template for G3P6 and G4P6

gBlock	Random7	ACACTTTGCTATGCCCGGGGATAAAATGGAACCGCAGTGGAACCATAGCCCGCGCTGCTTTGCGAGCCTGTGCTGCGGCGGCAGCCATACCATGATGGCGTGGAACCATGTGATTAGCTGGAAAGGCCGCGATCTGGTGATTGGCGTGAACCGCCATTGCGCGACCCCGCCGCATAGCCAGTTTTGGCATAACGCGTGGTGGCAGGGCTTTATTAAACATGTGATTGAAACCCCGCGCCTGGGCAACATGGCGAAAAACATGCAGTGCTTTGCGGCGTGCGCGCTGGCGGTGGCGTTTCCGGTGGATATTAGCCAGCTGGGAGGAGGTAAACCTATTCCTAATCCCGGGGGCAGCGTGATTGTGCTGGATTGGGTGCAGGGCACCAACCTGGTGCATAAACAGCATAACACCATTAACCGCCGCCATCATCATAGCCATCAGGAAATGTATCCGTGGCCGACCGTGTTTGAAATGCATGGGAACCGCATTATTGAAGAAGGAGGACGTGATGTCTTC	template for G3P7 and G4P7
gBlock	Random8	ACACTTTGCTATGCCCGGGCGCGGCAAATGGATGATGTGCAACACCCATATGGCGCATTGGAACACCTATTTTTGGGATGGCAGCGCGGTGCATCTGACCGATGATTTTTATCGCAACGGCCCGGCGAAAAGCTATAACCTGTTTATGGTGCAGAACCATAAAGAAAGCCGCCATAACTATAAAGTGTGCTTTTTTTGCTATCTGATTACCACCTATATTACCATTGCGAAACATAAACGCATGAACGAAAACTATTGGTGGATGGCGCAGGTGTATCTGAAATTTGTGCGCTGGCATGCGCGCAACTGCTGCTATGCGGGAGGAGGTAAACCTATTCCTAATCCCGGGCATGTGCCGAAATGGTGGTATAAAGGCTTTGATTGGACCACCCAGGTGTGGCCGTATGCGGCGATGCTGGGCTTTATTAACGCGCATCATCTGGATACCGTGATGATTAAAATTCATCTGGGCGCGTTTCATAACTGCGATTGGGTGGAAGGAGGACGTGATGTCTTC	template for G3P8 and G4P8
gBlock	Random9	ACACTTTGCTATGCCCGGGGTGATGCAGGGCGATACCTGGTGGGAATGCGAACCGAGCGAAGCGGAAATTCAGATGCTGTATTGGCCGTGGGGCAGCCAGAAAGATCCGATTGATTGGGCGTATCTGTGCGATACCTGGAAATATACCGGCGATCTGTGCAGCGGCGGCCCGGAACAGCCGGATGAACATCGCATTCATGATGCGATTGGCCGCGCGTTTTATCGCCCGTGCCCGAGCCTGAACATGTATTATCTGAGCCAGCGCTGGGCGATTTTTGATACCCATAACAGCCTGGCGGCGGGCAGCTATTGCTTTATGGGAGGAGGTAAACC	template for G3P9 and G4P9

		TATTCCTAATCCCGGGGTGACCACCTTTAAACTGTGGGCGAAAGCGCTGGTGGCGTTTATGTATGATGCGGATCATCATGTGAACGATTTTCTGCCGACCCTGTATCGCGTGTATACCACCATGAACATTTGGCATTTTAAACATAAATGCCCGTGCACCAGCTATGGAGGACGTGATGTCTTC	
gBlock	Random10	ACACTTTGCTATGCCCGGGACCGGCCGCCAGGTGACCGAAGTGACCGTGTGGCATGCGCTGACCACCATTTGCGGCATTAGCGAACTGGAATTTACCTATGGCGCGTGCCCGATGTGGGAAAACATGGAACTGGAAAAATTTAGCGGCAACGTGTGCTATGAACTGCAGGATCATTGCTTTTGCGATTGGTGGCAGTATACCGAACGCTGCCTGGAAAACCTGCCGATGATTGAACTGCCGATTCAGTGGAAACCGTTTACCCTGCATGAATGGTGGATTATTGGCCGCTGCCCGCTGACCATTATGAACAGCTGGGCGGGAGGAGGTAAACCTATTCCTAATCCCGGGGATGATGATGATGAACTGCTGCGCCTGTGCGATAACATTACCTTTTTTATGATGTGCATTCATGAATTTACCATGAAACCGTGGTTTAAAACCATTTGGTTTCTGATGTGCTGGAACGCGTTTCTGAACCAGGGCAGCAATAGCACCCATGGAGGACGTGATGTCTTC	template for G3P10 and G4P10
primer	100mer_F	ACACTTTGCTATGCCCGGG	amplifying group 4 random small proteins
primer	100mer_R	GGGATTAGGAATAGGTTTACCTCCTCC	amplifying group 4 random small proteins
primer	50mer_F	GAGGTAAACCTATTCCTAATCCCGGG	amplifying group 3 random small proteins
primer	50mer_R1	AGGAATAGGTTTACCTCCTCCCCATTTCGCGCGATGGC	amplifying G3P1
primer	50mer_R2	AGGAATAGGTTTACCTCCTCCGTTCTGTTCATAGCTATCATCGCGG	amplifying G3P2
primer	50mer_R3	AGGAATAGGTTTACCTCCTCCATACATGCACATCGCATGCAG	amplifying G3P3
primer	50mer_R4	AGGAATAGGTTTACCTCCTCCCACAAAATATTCCGGCCACGC	amplifying G3P4
primer	50mer_R5	AGGAATAGGTTTACCTCCTCCATGTTTCGCCACGCTATACTGC	amplifying G3P5
primer	50mer_R6	AGGAATAGGTTTACCTCCTCCATAAATAATCTGCCAAATGGTCGGAAACG	amplifying G3P6
primer	50mer_R7	AGGAATAGGTTTACCTCCTCCTTCTTCAATAATGCGGTTCCCATGC	amplifying G3P7
primer	50mer_R8	AGGAATAGGTTTACCTCCTCCTTCCACCCAATCGCAGTTATGAAA	amplifying G3P8

primer	50mer_R9	AGGAATAGGTTTACCTCCTCCATAGCTGGTGCACGGGCAT	amplifying G3P9
primer	50mer_R10	AGGAATAGGTTTACCTCCTCCATGGGTGCTATTGCTGCCCT	amplifying G3P10
primer	G1P6_opti_F	ATCCCGGGCTGTCTATGTCTATCTGCATGCGTCCGAAAGGAGGAGGTAAACCTATTCCTA	amplifying G1P6 with *E.coli* optimized codon
primer	G1P6_2X_opti_F	ATCCCGGGCTGTCTATGTCTATCTGCATGCGTCCGAAACTGTCTATGTCTATCTGCATGCGTCCGAAAGGAGGAGGTAAACCTATTCCTA	amplifying G1P6_2X with *E.coli* optimized codon
primer	cvi_cvaC_pBAD_F	GGTACCAGGAGGAAACGATGGATAGAAAAAGAACAAAATTAGAGTTGTTATTTGC	first cloning of *cvi* and *cvaC* into pBAD180-Km
primer	pBAD_cvi_F	GGTGGTGAATTCAGGAGGAAACGATGGATAGAAA	amplifying *cvi*
primer	pBAD_cvi_R	GCGTGGTACCTCATTTAGAGTCAGAGTTC	amplifying *cvi*
primer	pBAD_cvaC_F	GGTGGTGGTACCATGAGAACTCTGACTCTAAAT	amplifying *cvaC*
primer	pBAD_cvaC_R	GGCGGCGTCGACTCTAGATTATAAACAAACATCACT	amplifying *cvaC*
primer	MccV_V5_R	CAGGTCGACTTAAGTAGAATCTAAACCTAGGAGAGGATTAGGAATAGGTTTACCTCCTCCTAAACAAACATCACTAAGATTATTTGGACT	to generate MccV_V5
primer	CvaAB_F	ATATCTAGATTTCAGTGCAATTTATCTCTTCAAATGTAGCACCTGAAGTCAGCCCCATACGATATAAGTTGTAATTCTCATGTTTGACAGCTTATCATCGATAAGCTTTAATGCGGTAGTTTATCACAGTTAAATTGCTAACGCAGTCAGGCACCGTGTAGGAGGAAACGATGTTTCGCCAGGATGCTTTAGAAAAC	amplifying *cvaA*/*cvaB* with pTc promoter
primer	CvaAB_R	ATACCTGAGGGTATTATTTAATATAAGAAAGAACAGTTATTGGACAATCCAC	amplifying *cvaA*/*cvaB*
primer	CvaAB_F2	ATAGAGCTCTGGGTACCCGGGGATCCTCTAGAGTCGACAGGAGGAAACGATG	amplifying *cvaA*/*cvaB* to construct pSK03
primer	CvaAB_R2	ATAGCATGCCTGCAGTTAAATAGAAATAACTC	amplifying *cvaA*/*cvaB* to construct pSK03
primer	EGF_strep_R	ATAGTCGACTTATTTTTCGAACTGCGGGTGAGACCATCCTCCGCGCAGTTCCCACCATTTCAGATC	to generate EGF_strep
primer	Eglin C_strep_R	ATAGTCGACTTATTTTTCGAACTGCGGGTGAGACCATCCTCCGCCCACATGCGGCACATGGTT	to generate EglinC_strep

**cvaC*: encodes microcin V (MccV)

Table S5. Plasmids

Name	Description	Usage
pACYC184	Cm^{R}, Tet^{R}	Backbone plasmid
pBAD18-Km	Kan^{R}	Backbone plasmid
pMMB67EH	Amp^{R}	Backbone plasmid
pBR322	Amp^{R}	Template
pHK22	Amp^{R}	Template
pSK00	pBAD18-Km derived plasmid, Kan^{R}	POI expression
pSK01	pACYC184 derived plasmid, Cm^{R}	CvaA/CvaB expression
pSK02	pACYC184 derived plasmid, Cm^{R}	CvaA/CvaB C32S expression
pSK03	pMMB67EH derived plasmid, Amp^{R}	POI, CvaA/CvaB expression
pSKP00	pBAD18-Km derived plasmid, Kan^{R}	Cvi, MccV expression
pSKP01	pBAD18-Km derived plasmid, Kan^{R}	Cvi, MccV_V5 expression
pSKP02	pBAD18-Km derived plasmid, Kan^{R}	G1P1 expression
pSKP03	pBAD18-Km derived plasmid, Kan^{R}	G1P2 expression
pSKP04	pBAD18-Km derived plasmid, Kan^{R}	G1P3 expression
pSKP05	pBAD18-Km derived plasmid, Kan^{R}	G1P4 expression
pSKP06	pMMB67EH derived plasmid, Amp^{R}	G1P5 expression
pSKP07	pBAD18-Km derived plasmid, Kan^{R}	G1P6 expression
pSKP08	pBAD18-Km derived plasmid, Kan^{R}	G1P7 expression
pSKP09	pBAD18-Km derived plasmid, Kan^{R}	G1P8 expression
pSKP10	pBAD18-Km derived plasmid, Kan^{R}	G1P9 expression
pSKP11	pBAD18-Km derived plasmid, Kan^{R}	G1P10 expression
pSKP12	pBAD18-Km derived plasmid, Kan^{R}	G2P1 expression
pSKP13	pBAD18-Km derived plasmid, Kan^{R}	G2P2 expression
pSKP14	pBAD18-Km derived plasmid, Kan^{R}	G2P3 expression
pSKP15	pBAD18-Km derived plasmid, Kan^{R}	G2P4 expression
pSKP16	pBAD18-Km derived plasmid, Kan^{R}	G2P5 expression
pSKP17	pBAD18-Km derived plasmid, Kan^{R}	G2P6 expression
pSKP18	pBAD18-Km derived plasmid, Kan^{R}	G2P7 expression
pSKP19	pBAD18-Km derived plasmid, Kan^{R}	G2P8 expression
pSKP20	pBAD18-Km derived plasmid, Kan^{R}	G2P9 expression
pSKP21	pBAD18-Km derived plasmid, Kan^{R}	G2P10 expression

pSKP22	pBAD18-Km derived plasmid, Kan^R	G3P1 expression
pSKP23	pBAD18-Km derived plasmid, Kan^R	G3P2 expression
pSKP24	pBAD18-Km derived plasmid, Kan^R	G3P3 expression
pSKP25	pBAD18-Km derived plasmid, Kan^R	G3P4 expression
pSKP26	pBAD18-Km derived plasmid, Kan^R	G3P5 expression
pSKP27	pBAD18-Km derived plasmid, Kan^R	G3P6 expression
pSKP28	pBAD18-Km derived plasmid, Kan^R	G3P7 expression
pSKP29	pBAD18-Km derived plasmid, Kan^R	G3P8 expression
pSKP30	pBAD18-Km derived plasmid, Kan^R	G3P9 expression
pSKP31	pBAD18-Km derived plasmid, Kan^R	G3P10 expression
pSKP32	pBAD18-Km derived plasmid, Kan^R	G4P1 expression
pSKP33	pBAD18-Km derived plasmid, Kan^R	G4P2 expression
pSKP34	pBAD18-Km derived plasmid, Kan^R	G4P3 expression
pSKP35	pBAD18-Km derived plasmid, Kan^R	G4P4 expression
pSKP36	pBAD18-Km derived plasmid, Kan^R	G4P5 expression
pSKP37	pBAD18-Km derived plasmid, Kan^R	G4P6 expression
pSKP38	pBAD18-Km derived plasmid, Kan^R	G4P7 expression
pSKP39	pBAD18-Km derived plasmid, Kan^R	G4P8 expression
pSKP40	pBAD18-Km derived plasmid, Kan^R	G4P9 expression
pSKP41	pBAD18-Km derived plasmid, Kan^R	G4P10 expression
pSKP42	pBAD18-Km derived plasmid, Kan^R	codon-optimized G1P6 expression
pSKP43	pBAD18-Km derived plasmid, Kan^R	G1P6_2X expression
pSKP44	pBAD18-Km derived plasmid, Kan^R	G3P2_2X expression
pSKP45	pBAD18-Km derived plasmid, Kan^R	Pediocin PA-1 expression
pSKP46	pBAD18-Km derived plasmid, Kan^R	α-factor
pSKP47	pBAD18-Km derived plasmid, Kan^R	Eglin C expression
pSKP48	pBAD18-Km derived plasmid, Kan^R	EGF expression
pSKP49	pMMB67EH derived plasmid, Amp^R	Pediocin PA-1, CvaA/CvaB expression
pSKP50	pMMB67EH derived plasmid, Amp^R	Pediocin PA-1 expression
pSKP51	pBAD18-Km derived plasmid, Kan^R	EGF_strep expression
pSKP52	pBAD18-Km derived plasmid, Kan^R	Eglin C_strep expression
